# Proteomic elucidation of the targets and primary functions of the picornavirus 2A protease

**DOI:** 10.1016/j.jbc.2022.101882

**Published:** 2022-03-31

**Authors:** Artem A. Serganov, Yael Udi, Milana E. Stein, Valay Patel, Peter C. Fridy, Charles M. Rice, Mohsan Saeed, Erica Y. Jacobs, Brian T. Chait, Michael P. Rout

**Affiliations:** 1 Laboratory of Cellular and Structural Biology, The Rockefeller University, New York, New York, USA; 2 Laboratory of Virology and Infectious Disease, The Rockefeller University, New York, New York, USA; 3 Department of Biochemistry, Boston University School of Medicine, Boston, Massachusetts, USA; 4 National Emerging Infectious Diseases Laboratories, Boston University School of Medicine, Boston University, Massachusetts, USA; 5 Laboratory of Mass Spectrometry and Gaseous Ion Chemistry, The Rockefeller University, New York, New York, USA; 6 Chemistry Department, St John's University, Queens, New York, USA

**Keywords:** picornavirus, protease, plus-stranded RNA virus, nuclear pore, nuclear transport, proteomics, mass spectrometry, CVB3, Coxsackievirus B3, FISH, fluorescent *in situ* hybridization, HTLV-1, human T-cell leukemia virus type 1, Kaps, karyopherins, MX2, myxovirus resistance 2 protein, NES, nuclear export signal, NLS, nuclear localization signal, NPC, nuclear pore complex, PFA, paraformaldehyde

## Abstract

Picornaviruses are small RNA viruses that hijack host cell machinery to promote their replication. During infection, these viruses express two proteases, 2A^pro^ and 3C^pro^, which process viral proteins. They also subvert a number of host functions, including innate immune responses, host protein synthesis, and intracellular transport, by utilizing poorly understood mechanisms for rapidly and specifically targeting critical host proteins. Here, we used proteomic tools to characterize 2A^pro^ interacting partners, functions, and targeting mechanisms. Our data indicate that, initially, 2A^pro^ primarily targets just two cellular proteins: eukaryotic translation initiation factor eIF4G (a critical component of the protein synthesis machinery) and Nup98 (an essential component of the nuclear pore complex, responsible for nucleocytoplasmic transport). The protease appears to employ two different cleavage mechanisms; it likely interacts with eIF3L, utilizing the eIF3 complex to proteolytically access the eIF4G protein but also directly binds and degrades Nup98. This Nup98 cleavage results in only a marginal effect on nuclear import of proteins, while nuclear export of proteins and mRNAs were more strongly affected. Collectively, our data indicate that 2A^pro^ selectively inhibits protein translation, key nuclear export pathways, and cellular mRNA localization early in infection to benefit viral replication at the expense of particular cell functions.

Picornaviruses are small, nonenveloped RNA viruses that, like other viruses, subvert their hosts’ cellular functions to propagate (1). These functions can include host protein synthesis (2), intracellular protein and nucleic acid transport (3), and innate immune responses (4). The genus *Enterovirus* includes most of the human disease-causing picornaviruses, which are diverse and whose infections cause a variety of impactful clinical symptoms including diseases of the central nervous system leading to paralysis (poliovirus), respiratory system (rhinovirus), and heart (Coxsackievirus B3—CVB3), among many others (5). Further elucidating the mechanisms of picornaviral replication, and specifically how picornaviruses subvert infected cells and which host proteins are manipulated, would prove invaluable toward the development of novel treatment strategies for picornaviral infections.

The picornaviral genome is a positive-sense, single-stranded RNA that is translated by host ribosomes into a polyprotein comprising capsid and nonstructural proteins. This polyprotein is co-translationally processed into individual components by two virally encoded proteases, 2A (2A^pro^) and 3C (3C^pro^). Protease 2A^pro^ makes an initial cleavage during translation to separate the viral capsid proteins (VP1-4) from the remaining polyprotein (6), while 3C^pro^ makes all subsequent polyprotein cleavages to liberate individual viral proteins. Many of the translated viral proteins still have incompletely defined roles. For example, the 2A and 3C proteases have been reported to also cleave dozens of different host proteins, ranging from translation factors, RNA binding proteins, nuclear pore complex (NPC) components involved in protein and nucleic acid trafficking, innate immune proteins, and many others (7, 8, 9, 10, 11, 12). It is hypothesized that both proteases, especially 2A^pro^, play a critical role for promoting picornaviral replication and attenuating the host antiviral response. Given 2A^pro^’s potential importance in subverting host function, its cleavage targets and catalytic function have been intensively studied by virologists in several model systems and in a diverse array of picornaviruses, but the mechanism(s), temporal progression, and effects of protease activity on host protein complexes remain unclear (7, 8, 9, 11, 13). Here, we pursued a biochemical and proteomic approach to explore the function of 2A^pro^ from Coxsackievirus B3, one of the most widely studied pathogenic picornavirus species. We focused our efforts specifically on the protease, outside of an infectious context where a plethora of virally induced changes could complicate our analyses. We present evidence supporting the idea that, in the earliest stages of infection, 2A^pro^ targets a small number of specific host targets and over the course of infection eventually broadens its target list as more viral protease accumulates through continuous polyprotein translation.

## Results

### Affinity capture identifies Nup98-Rae1 and the eIF3 complex as primary binding partners of 2A^pro^

**Figure 1:**
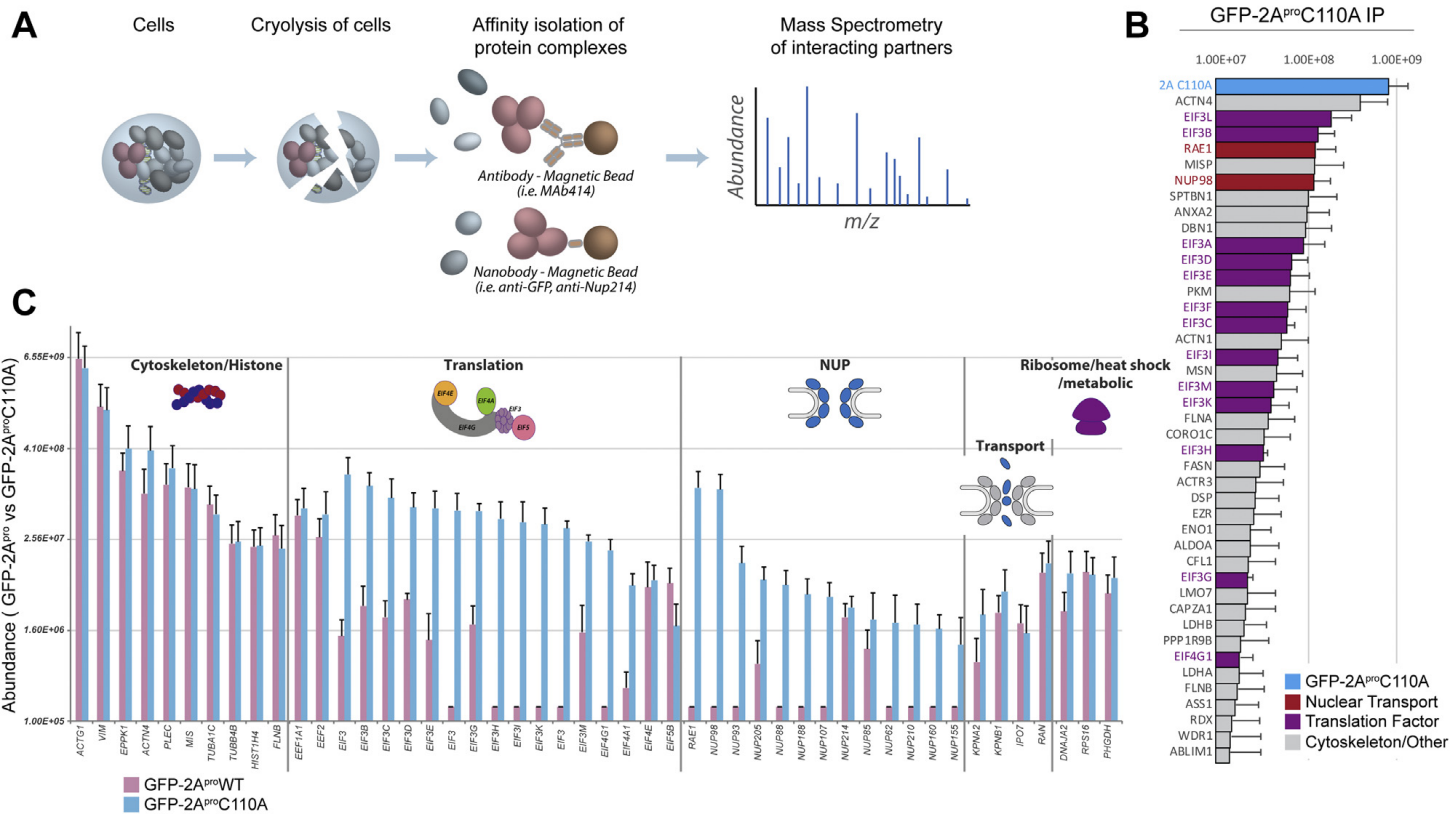
**Biochemical isolation of 2A**^**pro**^**interactome and binding specificity to Nup98**. *A*, pipeline for affinity isolation of 2A^pro^ interacting partners. *B*, GFP-2A^pro^C110A interactome identified by MS (protein identity on Y-axis, peptide abundance on X-axis). *C*, side-by-side comparisons of protein interactomes of WT and mutant 2A^pro^ affinity capture experiments. Protein identities are on the X-axis, organized by category of proteins (such as protein translation or Nup), with the Y-axis plotting peptide abundance. It is apparent that proteolytic activity causes a decrease in peptide abundance of a number of translation and transport related proteins. Adapted from Xiang Y. *et al* (2020).

We have previously developed an efficient affinity capture pipeline that is effective for a diversity of proteomic studies (14, 15, 16), one that has also been applied successfully to the study of viral–host interactions during infection (Fig. 1*A*) (17, 18, 19, 20). For our proteomic studies of picornaviral 2A^pro^, we established stable HeLa cell lines (using the PiggyBac system), allowing expression under a doxycycline-inducible promoter of GFP-tagged forms of WT 2A^pro^ or its catalytically inactive variant carrying a Cys-110-Ala mutation (2A^pro^C110A) (21). The GFP tag served to facilitate detection of protease production; moreover, it allowed for efficient affinity capture of catalytically active and inactive proteases and their interacting partners. Cells expressing the GFP tag without attached protease served as an additional control in these experiments.

**Figure 2:**
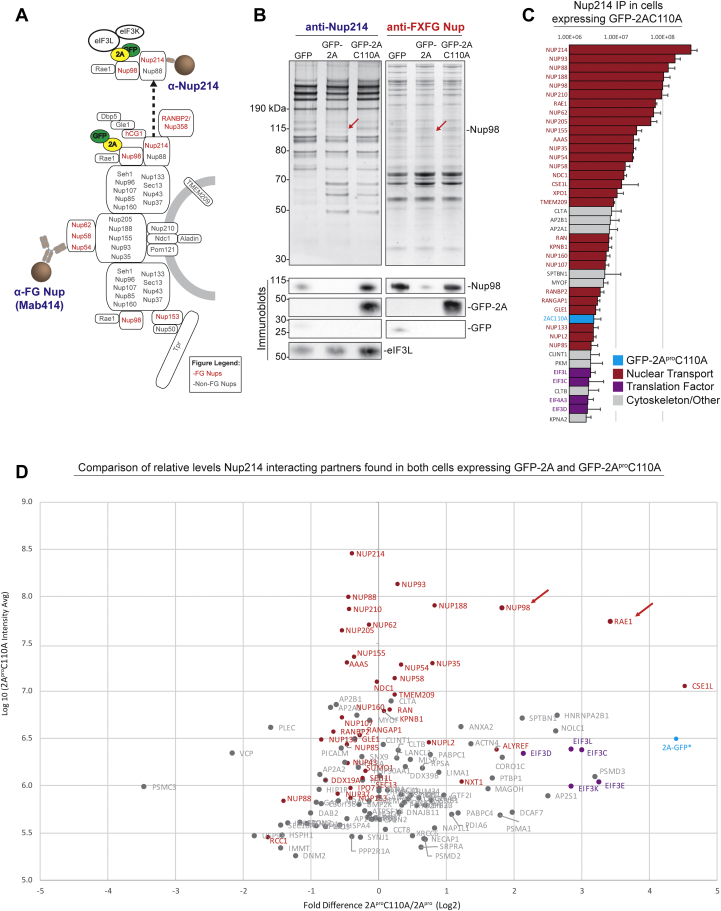
**Nup98 and eIF3 are primary interacting partners of the 2A protease during FG-Nup affinity capture**. *A*, diagram of mammalian NPC with immunoprecipitation targets identified by specific antibody or nanobody (*blue* —Nup214, *red—*mAB414 specific for FG Nups). The protein complex (Nup214-Nup98-2A^pro^C110A-eIF3L) proposed to be isolated during Nup214 affinity capture is shown above. *B*, affinity capture of Nup214, a cytoplasmic FG-Nup, and FXFG Nups reveals association with many NPC components. IP elutions were clarified by SDS-PAGE and analyzed by Silver stain (*top panels*) and immunoblotting (*bottom panels*). Nup214 and FG Nup IPs suggest 2A^pro^ specifically cleaves Nup98. Immunoblot analysis suggest that immunopurifying GFP-2A^pro^C110A, whether directly or indirectly (Nup214 IP) also correlates to an enrichment of eIF3L in eluates. *C*, top interacting partners identified by MS during Nup214 affinity capture (protein identity on Y-axis, peptide abundance on X-axis). *D*, affinity capture of Nup214 in the biological context of 2A and 2A^pro^C110A expression reveals enrichment of Nup98, Rae1, and eIF3L, among other proteins, during 2A^pro^C110A expression. Nup98 and Rae1 enrichment are due to cleavage of Nup98 in the 2A^pro^ expressing cells and subsequent loss of Rae1 interaction to the Nup complex, while eIF3L and eIF3K are coprecipitating with 2A^pro^C110A. The X-axis shows Log_2_ transformed fold-difference in label-free quantified MS abundance between datasets and the Y-axis measures 2A^pro^C110A mean intensity.

It is well established that 2A^pro^ shuts down host translation through eIF4G cleavage during picornaviral infections (10, 22, 23), which is presumed to reduce the use of host energy and available resources for housekeeping functions and diminish cellular antiviral responses to infection (24). Ectopic expression of picornavirus 2A^pro^ also quickly depletes cellular eIF4G levels and stops host translation (25, 26). This translational arrest creates a situation where expression of 2A^pro^ also ceases, thereby limiting its detectable accumulation in these cells. Therefore, we used the cleavage of 2A^pro^ targets as a proxy to assay the expression of WT 2A^pro^. Among the reported substrates of 2A^pro^ (see also below) are Nup98 and eIF4G, which were indeed rapidly depleted following doxycycline treatment of HeLa cells expressing GFP-2A^pro^, but as expected not those expressing GFP or GFP-2A^pro^ C110A (Figs. S1 and S2) (27, 28). Having confirmed the expression of our constructs, we grew the doxycycline-treated cells to preparative quantities and subjected them to cryogenic lysis, producing homogenous cell powders that can be stably stored long term and can be weighed out into tubes for affinity capture experiments (16), providing consistency across experiments. We then affinity captured the GFP-tagged proteases from these lysates, together with associating proteins (Fig. 1*B*). GFP-2A^pro^C110A was successfully affinity captured with significantly more interacting proteins than the GFP control or GFP-2A^pro^, as visualized by SDS-PAGE (Fig. S3). Notably, we were able to affinity capture WT GFP-2A^pro^, although the protein levels were considerably lower than for GFP or GFP-2A^pro^C110A. This is expected, since host proteins that are cleaved by 2A^pro^ are degraded and no longer available for co-purification; moreover, WT 2A^pro^ activity shuts down host translation, preventing replenishment of targeted proteins. However, in the absence of protease cleavage and subsequent product release, intermediate complexes consisting of substrate bound to the inactive protease may be stabilized. For these reasons, we focused our affinity capture efforts on GFP-2A^pro^C110A to better reveal the protease binding partners and cleavage targets. We analyzed affinity capture eluates by immunoblotting and mass spectrometry (MS) (Figs. 1*C*, 2*A*, S4 and Table S1).

We ranked proteins in the GFP-2A^pro^C110A affinity capture dataset by protein abundance and compared fold enrichments of proteins found in both 2A^pro^ and 2A^pro^C110A datasets compared to the GFP control (Fig. S5). We plotted the top ∼40 interacting proteins by label-free protein abundance determination (Fig. 1*B*). Identified proteins were broken down into three categories: Nups (red), translation initiation factors (purple), and cytoskeletal and other proteins (gray), the latter being mainly composed of residual contaminants (29). The top groups of interacting proteins were dominated by Nup98-Rae1 and almost the entire eIF3 complex. Nup98 and Rae1 were detected with an approximately 1-to-1 protein stoichiometric ratio, based on label-free MS quantitation. Given these data, it is likely that 2A^pro^C110A is binding one of these two proteins during affinity capture. Since Nup98 is a known cleavage target (30), we suspect 2A^pro^C110A is forming a direct interaction with this Nup, prior to cleaving it (Fig. S6*A*). Rae1 is well characterized as a shuttling mRNA export factor directly bound to Nup98 through the latter’s WD40-GLEBS motif (31, 32). Considering the strong affinity between the GLEBS domain of Nup98 and WD40 motifs of Rae1 (32), it is likely that Rae1 is pulled down with Nup98, as an ‘indirect’ binder of 2A^pro^C110A (Fig. S6*B*).

Interestingly, we also observed comparably high levels of eIF3L and eIF3B in the MS data analysis, as well as other eIF3 complex proteins (Fig. 1, *B* and *C*). In all, we detected 12 out of 13 proteins that form the eIF3 complex (33), and these proteins were almost entirely in the top 20 most prevalent proteins of a 2A^pro^C110A affinity capture. Unexpectedly, eIF4G was present at 10-fold lower levels than these EIF3 proteins, even though eIF4G is known to be one of the primary cleavage targets of 2A^pro^ and so might be expected to be abundant (34, 35, 36). We considered whether these observed ratios were simply reflecting the cellular protein abundance of eIF3 and eIF4G; however, MS analysis of our cell lysates (data not shown) and published work (37) indicates that there is 2 to 3 fold as much eIF4G as eIF3L and the other eIF3 proteins, making this an unlikely explanation. Given the interactomic data, specifically the high levels of eIF3 proteins compared to eIF4G, the predominance of eIF3L in multiple rounds of affinity captures and the known stability of the eIF3 complex between rounds of translation (33, 38, 39), we hypothesized that eIF3L acts as a primary binding site and staging partner for 2A^pro^, from which the protease cleaves eIF4G during translation initiation (Fig. S6*C*). While we tentatively propose eIF3L to be the predominant binding partner for 2A^pro^, it is plausible that the protease makes additional interactions with other eIF3 complex components *in vivo*.

Despite limitations resulting from the cleavage of targets and shut down of host translation for the WT protease, we were nevertheless able to glean useful results from the affinity capture of WT GFP-2A^pro^. Nup98 was not detected in the 2A^pro^ dataset, almost certainly because nearly all Nup98 are cleaved within cells expressing the protease (Fig. S1). Rae1 was not detected in 2A^pro^ affinity capture data either, again consistent with it only being present in the 2A^pro^C110A data as the primary interacting partner of Nup98. The absence of other Nups from the 2A^pro^ affinity capture data is not surprising, as they are likely to be second and third order interactors of Nup98 as part of the NPC. We also observed small amounts of a number of eIF3 complex proteins, presumably because amongst them are binding targets of the protease (above), but they are not themselves its primary cleavage targets. We plotted the top 20 most enriched proteins in the 2A^pro^C110A dataset compared to 2A^pro^ MS data, and unsurprisingly, the list consists of almost entirely NPC and eIF3 components (Fig. S5), underscoring the highly specific targeting of these over all other cellular components.

### Assessing 2A^pro^ cleavage of NPC components

Our 2A^pro^C110A affinity capture experiments detected Nup98 bound to the catalytically inactive protease, with Rae1 bound to Nup98. However, both Nup98 and Rae1 were largely depleted from 2A^pro^ affinity capture experiments. To determine whether Rae1 was being cleaved similarly to Nup98, we assayed our 2A^pro^-expressing cell line lysates by immunoblotting (Fig. S1). Indeed, 2A^pro^ expression led to nearly total depletion of Nup98, but no cleavage of Rae1. Since Rae1 is not proteolytically degraded by 2A^pro^, we infer that Rae1’s absence from 2A^pro^ affinity capture data is due to depletion of Nup98 and subsequent lack of primary binding partner for Rae1. Assaying eight other Nups by immunoblotting, we observed that 2A^pro^ did not affect other NPC components tested, suggesting a high degree of specificity for Nup98 (Fig. S1). eIF3L was also assayed by immunoblotting and was not found to be degraded. Since endogenous eIF3L levels are not affected by 2A^pro^ catalytic activity, this suggests that decreased levels of eIF3 components in the 2A^pro^ affinity capture data are likely caused by other factors, such as tertiary complex destabilization.

### Nup214 affinity capture during expression of WT or mutant 2A^pro^ validates protease interactions with Nup98 and eIF3

Our laboratories have developed Nup214-specific nanobodies that allow the affinity capture experiments of essentially the entire NPC. We thus affinity captured Nup214 and associated NPC proteins from cryolysed cells, utilizing our established stable WT or mutant protease expressing cell lines (Fig. 2*A*). Immunoblots demonstrate that Nup98 is captured during Nup214 affinity capture and that Nup98 is missing during WT 2A^pro^ expression, almost certainly due to proteolytic degradation (Fig. 2*B*). Furthermore, the protease was only detected in affinity captures from GFP-2A^pro^C110A expressing cells but not those expressing GFP-2A^pro^, further supporting the proposal for a direct interaction between 2A^pro^C110A and Nup98. Notably, eIF3L was also detected at much more elevated levels in the 2A^pro^C110A expressing cells (Fig. 2*B*). This increase in eIF3L level correlated with the associated affinity capture of 2A^pro^C110A, supporting the idea of a Nup98-2A^pro^C110A-eIF3L interaction. Given this observation, we propose that 2A^pro^ can bind Nup98 and eIF3L simultaneously, the former through the catalytic site and the latter potentially through another site (see also below).

The top 40 protein interactors of the Nup214 affinity capture during GFP-2A^pro^C110A production were ranked by label-free protein abundance and unsurprisingly revealed NPC and nuclear transport proteins as the top Nup214 associating partners (Fig. 2*C*). Nup98 was ranked fifth, and GFP-2A^pro^C110A was detected in the top 40. This reverse affinity capture thus provides further evidence for a direct interaction formed between Nup98 and the 2A^pro^. In addition, we detected eIF3 complex proteins with relatively similar protein abundances as 2A^pro^C110A, reinforcing the hypothesis of a direct 2A^pro^C110A–eIF3L association. We compared Nup214 affinity capture datasets under 2A^pro^ and 2A^pro^C110A expression to investigate interactomic profiles and look for any outlier protein enrichment and plotted these data as a volcano plot (Fig. 2*D*). A number of proteins were found to be more than 3-fold enriched in the 2A^pro^C110A affinity capture dataset, such as Nup98, Rae1, eIF3L, and eIF3K. It is not surprising that Nup98-Rae1 was highly enriched in the 2A^pro^C110A dataset compared to 2A^pro^ data, given that 2A^pro^ degrades and depletes Nup98. However, it was surprising to detect enrichment of eIF3 proteins in the Nup214 affinity capture with 2A^pro^C110A expressed, given these eIF3 proteins would effectively be fourth order interactions *via* the proposed binding order of Nanobody-Nup214-Nup98-2A^pro^C110A-eIF3 (Fig. 2*A* – top half). Attempts to dissect and confirm which eIF3 component (such as eIF3L or eIF3K) was definitively binding to 2A^pro^C110A (such as through chemical cross-linking, RNAi, or finding a chemical inhibitor of eIF3 complex formation) were unsuccessful, prompting the need for future larger scale biochemical studies to validate binding interactions. In a similar manner to the Nup214 affinity captures, we also assayed 2A^pro^C110A association with Nup98 *via* capture of the NPC through a different bait, using the FG nup-specific monoclonal antibody, MAb414. Again, Nup98 was found to be depleted during 2A^pro^ production (Fig. 2*B*), and GFP-2A^pro^C110A was found to cofractionate with NPCs. Overall, our data reveal that CVB3 2A^pro^ does not immediately target Nups other than Nup98 nor dramatically reorganize the structure of the NPC (though it may weaken its structure through loss of Nup98) and instead seemingly focuses on specifically depleting only Nup98—and through it, Rae1—from the NPC.

### Assessing 2A^pro^ cleavage specificity for Nup98 compared with other Nups

**Figure 3:**
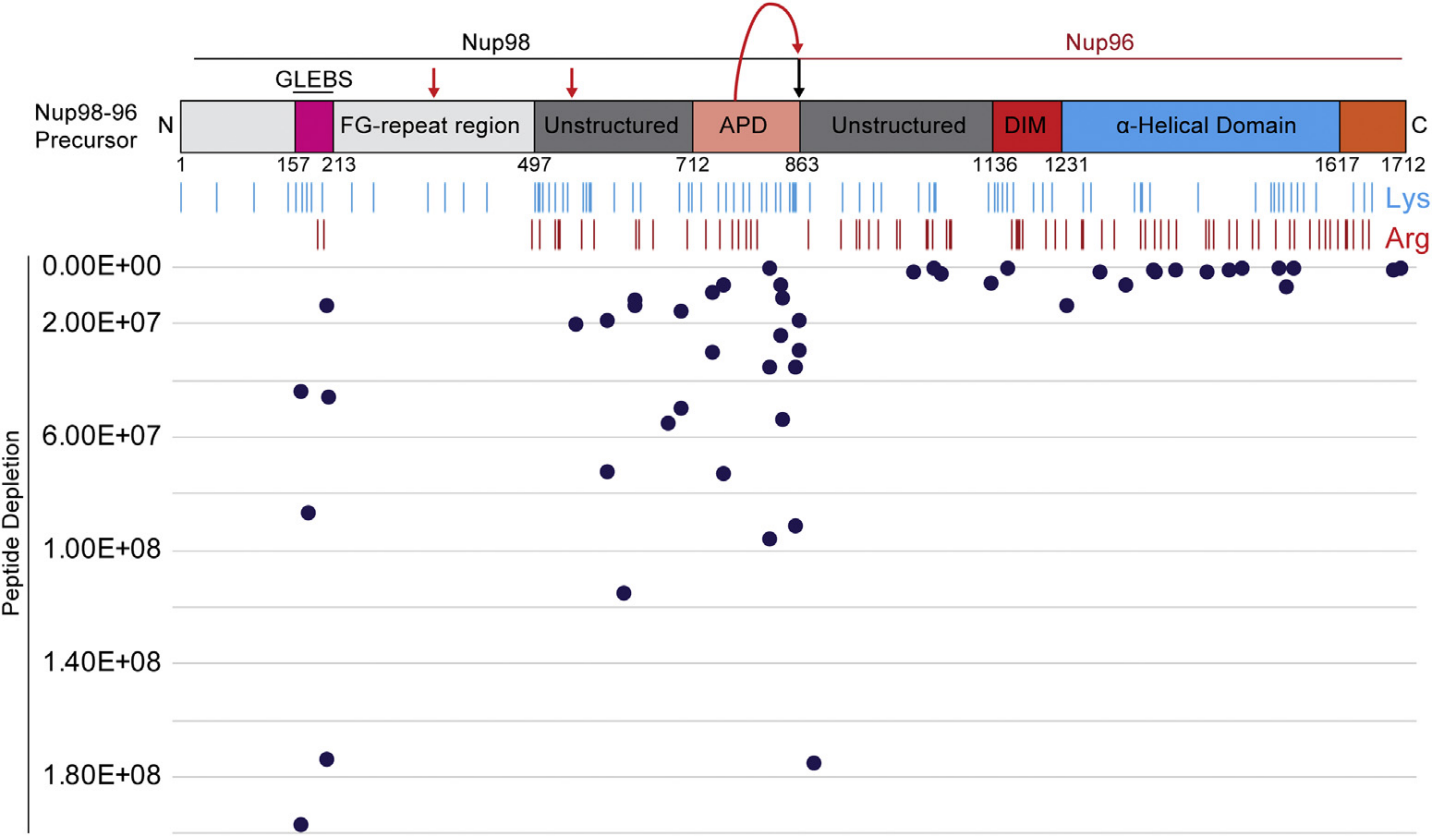
**Nup98 to 96 peptides plotted against secondary structure and assessed for depletion during 2A**^**pro**^**expression.** Nup98 is translated as a bicistronic fusion with 96 and separated into individual proteins by the autoproteolytic domain (APD). We plotted the label-free abundance of all Nup98 to 96 peptides identified by MS along the secondary structure of the fusion protein (X-axis) and observed depletion of Nup98 peptides originating only from its unstructured region specifically during 2A^pro^ expression as measured and plotted along Y-axis (peptides located further down the Y-axis are more depleted). *Red arrows* indicate putative cleavage sites for 2A^pro^.

Nup98 is normally produced as a bicistronic fusion protein that is separated into individual components by an autoproteolytic domain at the C terminus of Nup98 (Fig. 3) (40). After cleavage, Nup98 and Nup96 retain a binding interaction with each other *via* the C terminus of Nup98 which tethers it to the NPC’s outer ring Y-shaped complex (30). By analyzing depletion of Nup98–Nup96 peptides from the same experiments as in Figure 2, we can assess how specific 2A^pro^ proteolytic activity is or whether it spreads to adjoining Nups, such as Nup96. We plotted all identified Nup98-96 peptides along the length of the fusion protein (Fig. 3). We detected enormous depletion of Nup98 peptides within Nup214 affinity captures in the presence of 2A^pro^ along the entire length of Nup98; however, Nup96 peptide levels were unaffected (as were those of all the other Nups detected; not shown), which is consistent with the hypothesis that 2A^pro^ solely degrades Nup98 and not neighboring NPC components, despite their proximal availability.

### Nup98 degradation mediated by 2A^pro^ affects nuclear import and protein localization

**Figure 4:**
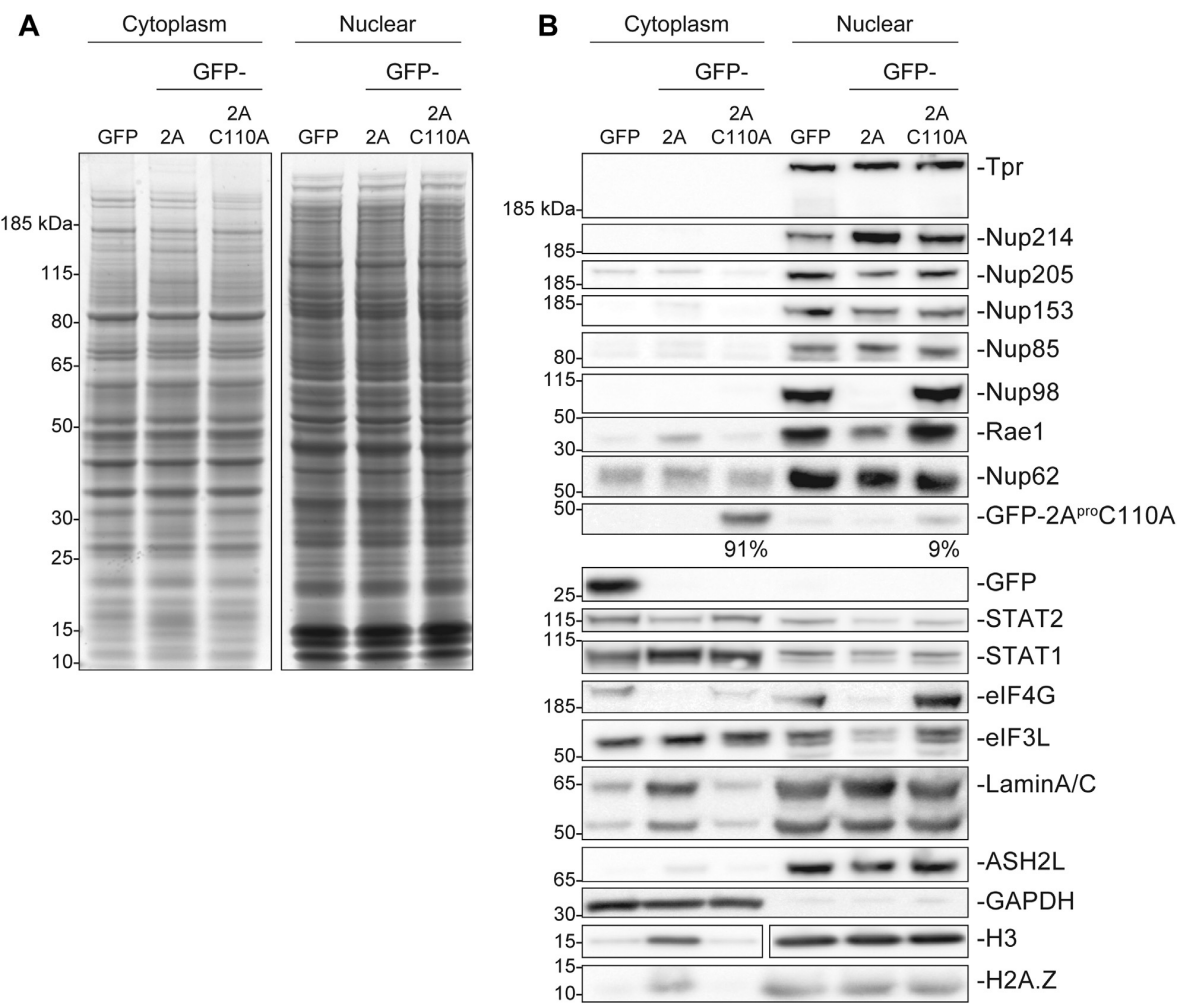
**Subcellular fraction of nuclear proteins during 2A**^**pro**^**expression.** HeLa cells induced to express GFP, or catalytically active or inactive 2A protease, were subcellularly fractionated into cytoplasmic and nuclear fractions. *A*, Coomassie stain of fractions from fractionation steps, including the cytoplasmic and nuclear fractions. *B*, Western blot analysis of nuclear markers (H3, lamin A/C, ASH2L), a cytoplasmic marker (GAPDH), a variety of NPC structural (Tpr, Nup205, Nup85) and FG Nups (Nup214, Nup153, Nup98, Nup62), interferon-related pathways (STAT1, STAT2), as well as GFP, eIF3L, and Rae1. The depletion of specific Nups in the nuclear fraction in response to 2A^pro^ expression is identified below the respective blot. Notably, Nup98 and Rae1 are significantly and specifically depleted due to 2A^pro^ activity. Nup98 is degraded while Rae1 dissociates from the NPC due to lack of GLEBS motifs otherwise found on Nup98. GFP-2A^pro^C110A can also be detected in the nuclear fraction as it associates through Nup98.

It is well established that during the course of picornavirus infection, 2A^pro^ expression directly affects nucleocytoplasmic protein and RNA transport (27, 12, 41, 42). The underlying hypothesis is that nuclear-localized proteins that promote picornaviral replication, such as by facilitating formation of an internal ribosome entry site for translation, need to relocalize to the cytosol where viral replication occurs. This relocalization is driven through targeting of NPC and nuclear transport components (43, 44), and our results here indicate these alterations may in fact be initially accomplished largely by specific targeting of just Nup98. We therefore investigated the functional effects of this 2A^pro^-mediated Nup98 degradation on nucleocytoplasmic transport. We used cellular fractionation to accurately and effectively separate the nuclear and cytoplasmic fractions of mammalian cell lines. HeLa cells producing the viral protease and controls were fractionated, and the fractions were assayed by SDS-PAGE and immunoblotting (Fig. 4). By Coomassie-stained SDS-PAGE, fraction samples from GFP, GFP-2A^pro^, and GFP-2A^pro^C110A producing cell lines looked very similar. (Fig. 4*A*). In order to analyze the cellular localization of specific proteins during 2A^pro^-mediated cleavage of Nup98, we immunoblotted the fractionation experiments against other NPC components, cell fraction markers, translation factors, transcription factors, and other proteins (Fig. 4*B*).

Our experiments confirmed that a cytosolic control protein, GAPDH (45), did not contaminate the nuclear fractions, while also confirming equal gel loading of cytoplasmic fraction samples (Fig. 4*B*). Two other proteins can serve as nuclear markers. The first is lamin A/C, a filamentous protein that localizes to the inner face of the nuclear envelope (and maintains nuclear structure there) (46). The second is ASH2L, a histone lysine methyltransferase (47). The 2A^pro^ expression seems to significantly increase lamin A/C accumulation in the cytosol. It is likely that 2A^pro^-mediated cleavage of Nup98 has inhibited specific transport pathways, such as Importin-α/β, that mediate the passage of newly translated lamin A/C proteins to the nucleus. Similar cytoplasmic accumulation can also be observed with histone H3, which is predicted to be shuttled by importin-αs, but mostly IPO4 (48, 49). The 2A^pro^ expression is also known to trigger apoptosis in cells, which may manifest in our fractionation experiment as cross-fraction contaminations from whole cells breaking up. We confirmed these apoptotic effects of 2A^pro^ expression in our system (Fig. S8) by analyzing cell lysates with apoptotic markers. The 2A^pro^ expression is observed to trigger PARP and caspase-3 cleavage (Fig. S8*A*). In addition, we assayed cell viability during 2A^pro^ expression as well as apoptotic induction *via* paclitaxel with an XTT assay (Fig. S8*B*). As expected, 2A^pro^ expression lowered cell viability at the same degree as paclitaxel. However, apoptosis-mediated contamination in our fractionations should be ubiquitous for all assayed proteins (due to overall apoptotic degradation of the nucleus and nuclear envelope), while our observations are specific to Rae1, lamin A/C, and histone proteins (and not, *e.g*., ASH2L and most nucleoporins), suggesting these specific mislocalizations are largely due to changes in cell transport states. Given our data, we suspect that Nup98 depletion has a minor but noticeable impact on nuclear import, resulting in some nuclear-localized proteins, such as lamin A/C and histones H3 and H2A.Z, partially mislocalizing to the cytoplasm during translation of GFP-2A^pro^ (Fig. 4*B*).

NPC components assayed by immunoblotting largely remained unchanged and in the nuclear fraction, consistent with our other results (above). Meanwhile, Nup98 is observed as totally degraded during 2A^pro^ expression, which unsurprisingly led to significant relocalization of Rae1 from the nuclear fraction to the cytosol (Fig. 4*B*). We observed a portion of total GFP-2A^pro^C110A localizing to the nucleus, likely due to direct interactions formed between 2A^pro^C110A and Nup98, whereas free GFP signal was entirely localized to the cytoplasm. We also observed a decrease of eIF3L in the nuclear fraction of 2A^pro^ producing cells and a corresponding increase in the cytoplasm, matching a similar pattern of re-localization observed with Rae1. While some eIF4G was found in the cytosol (likely associated with translationally active ribosomes), most was detected in the nuclear fraction, presumably associated with ribosomes along the cytoplasmic surface of the nucleus, as well as in a previously described nuclear pool (50). eIF4G is cleaved during 2A^pro^ expression, and we observed a total depletion of any nuclear-localized eIF4G. We also observed increased localization of eIF3L to the cytosol from the nuclear fraction in response to 2A^pro^ activity, implying that this relocalization arises from eIF4G cleavage and general translation shutdown. Finally, we observed a modest 2A^pro^-mediated relocalization of STAT1 to the cytosol, in agreement with recent published literature showing that SARS-CoV-2 Orf6 hijacks Nup98 function and similarly blocks STAT nuclear import (51, 52). STAT2 levels decreased during 2A^pro^ expression in both fractions, suggesting there may be a 2A^pro^-mediated mechanism during picornaviral infections to reduce this signaling pathway. Interestingly, quantitation of STAT1 and STAT2 localization during fractionation reveals that production of either GFP-2A^pro^ or GFP-2A^pro^C110A triggers a robust decrease of both STAT1 and STAT2; however, the catalytically active protease is more than twice as effective at STAT depletion (Fig. S9). Abrogating STAT1 and STAT2 localization to the nucleus, and subsequently affecting their function as transcription factors, is yet another strategy employed by viruses to prevent host cells from responding to infections (43, 51).

### Protease 2A induces localization changes of mRNA in cells

**Figure 5:**
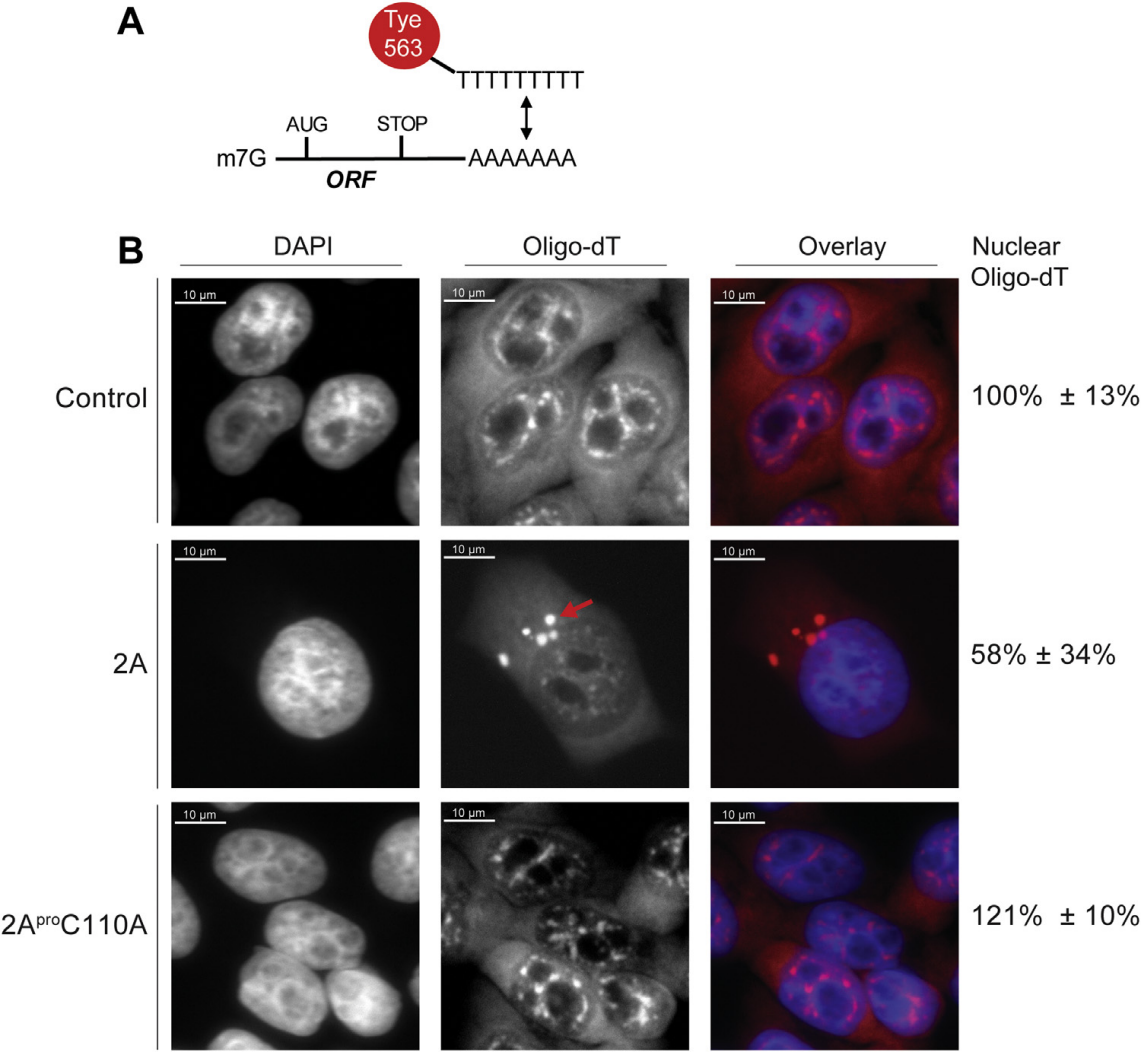
**2A**^**pro**^**-mediated cleavage of Nup98 results in mRNA relocalization formation**. *A*, Poly-T FISH was used to track mRNA export in cells. Tye563 is a red fluorescent dye that was conjugated to oligo-dT probes that bind the 3′ polyA tail of mRNA. *B*, 2A^pro^ expressing cells were observed to have less mRNA signal and mislocalization of RNA speckles. This mislocalization was directly tied to the catalytic activity of the 2A^pro^, as the mutant protein did not affect mRNA signal. During 2A^pro^ expression, RNA also seemed to localize to larger ‘speckles’ akin to P-bodies (*red arrow*). Signal reduction was assessed by measuring the total polyT FISH signal per cell and nuclei and comparing the average signal levels between each cell and across sample types.

We augmented our fractionation studies of nucleocytoplasmic localization of proteins with microscopy assays designed to further interrogate transport mechanics during 2A^pro^-mediated cleavage of Nup98 and disruption of associated transport pathways. We used an established protocol to generate a poly-T fluorescent *in situ* hybridization (FISH) stain to visualize mRNA localization (Fig. 5*A*) and assay the state of mRNA during production of GFP-2A^pro^ and degradation of Nup98. Expression of GFP had no effects on RNA localization, with the majority of signal localizing throughout the cytosol, and as small ‘speckles’ surrounding the perimeter within the nucleus (Fig. 5*B*). In contrast, GFP-2A^pro^ production and cleavage activity had a major impact on mRNA localization, with poly-T FISH signal forming fewer but slightly larger speckles reminiscent of P-bodies or stress granules along the nuclei and in the cytosol (53, 54, 55). Similar ‘speckles’ were observed during GFP fluorescence imaging of GFP-2A^pro^, suggesting the protease is also sublocalized within the granule-like structures (Fig. S7). Indeed, co-staining of poly-T FISH signal with G3BP1, a stress granule marker, shows colocalization of larger mRNA granules with G3BP1 signal, reinforcing the idea that GFP-2A^pro^ may mediate mRNA localization to stress granules (Fig. S10). Smaller ‘speckles’ of signal normally distributed throughout the nucleus mostly disappeared during GFP-2A^pro^ production. Furthermore, fluorescence quantification of overall mRNA levels reveals a roughly 2-fold 2A^pro^-mediated decrease in nuclear signal (Fig. 5 and Table S4).

### Analyzing effects of protease 2A on canonical nuclear transport with fluorescent nuclear localization signal and nuclear export signal reporters

We also augmented our studies with more specific assays designed to interrogate the actual transport mechanics during 2A^pro^-mediated cleavage of Nup98 and disruption of associated transport pathways. Nucleocytoplasmic transport of proteins depends on karyopherins (Kaps) that detect specific nuclear localization signals (NLSs) and nuclear export signals (NESs) on their cargo; Kaps can be broken down into two major categories: importins that bind NLS-cargo and transport it to the nucleus and exportins that bind NES-cargo in the nucleus and shuttle it to the cytosol. All Kaps interact directly with FG-repeats of Nups, with some Kaps preferring certain types of FG repeat Nups over others during transport; thus, destruction of Nup98 (a Nup carrying a particular type of FG repeat) might plausibly affect certain transport pathways more than others. By utilizing a series of fluorescent reporters each carrying different kinds of NLS or NES (recognized by different cognate Kaps) (Fig. S12*A*) in cells expressing either 2A^pro^ and 2A^pro^C110A, we gained insights into changes in nucleocytoplasmic transport induced by active 2A^pro^. Some canonical NLS and NES tags are sufficiently well characterized that we understand which transport factors mediate their transport; for instance, SV40 NLS is transported to the nucleus by importin α/β, while the PKI NES export operates through CRM1/XPO1 (56), and both of these import and export pathways have been reported to be impacted by 2A^pro^ expression and catalytic activity (57).

**Figure 6:**
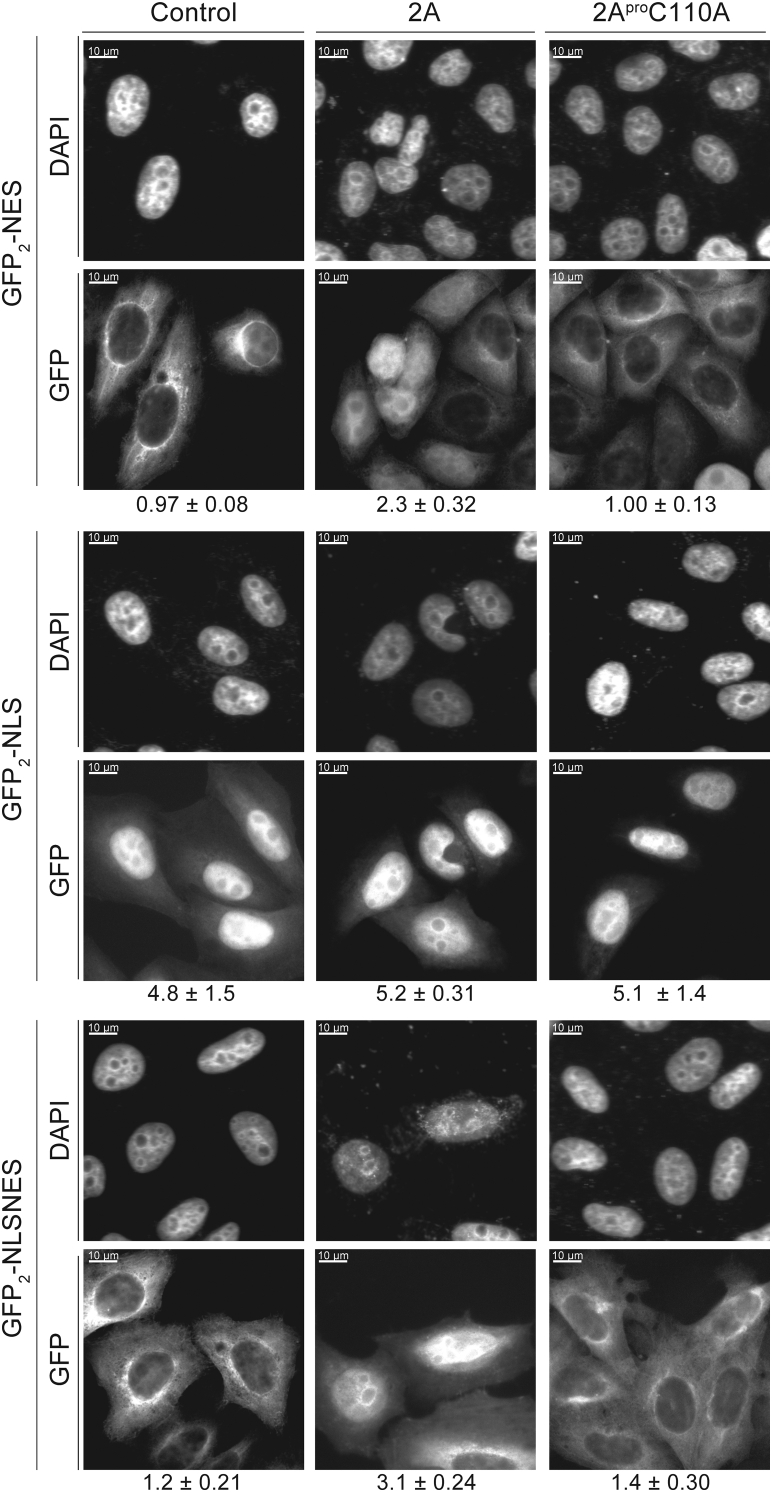
**GFP2-NLS/NES/NLSNES protein fusions are constitutively expressed in a HeLa stable cell line**. Cells were either untreated (control—unperturbed signal) or transfected to express GFP-2A^pro^ or GFP-2A^pro^C110A. Cells were imaged under DAPI and FITC filters, with DAPI showcasing nuclear localization, and FITC demonstrating GFP2 reporter localization during various protease production contexts. We calculated the ratio of nuclear signal over cytoplasmic signal for GFP2 localization and observed a marked increase in nuclear localization of GFP2-NES during proteolytic activity. NES, nuclear export signal; NLS, nuclear localization signal.

GFP_2_-NES is a tandem GFP–GFP construct with a C-terminal PKI NES that normally localizes to the cytosol, with higher intensity fluorescent signal around the nuclear rim (56). However, during 2A^pro^ expression, we observed a significant signal accumulation in the nucleus (Figs. 6 and S11). In contrast, 2A^pro^C110A expression had no impact on localization of the GFP reporter. Nup98 is a cofactor for CRM1-dependent protein export (58), rationalizing why Nup98 depletion by 2A^pro^ led to inhibition of CRM1-mediated export of the GFP_2_-NES reporter. By contrast, a GFP_2_ reporter with the SV40 NLS normally localizes to the nucleus, with weak signal in the cytoplasm. This cytoplasmic GFP signal increased slightly in cells expressing 2A^pro^ but not those expressing 2A^pro^C110A. This observation is consistent with the possibility that 2A^pro^ disrupts importin α/β function (57), although the effect is not as pronounced as that observed for the NES reporter. To further characterize the relative magnitude of 2A^pro^’s effect on NES-mediated export *versus* NLS-mediated import, we used a GFP_2_ reporter carrying both the NES and the NLS. Normally, this fusion protein localizes mostly to the cytosol with some nucleoplasmic signal. However, here it was found predominantly in the nucleus during 2A^pro^ production, indicating that its import pathway is less affected than its export pathway by Nup98 depletion (59). Collectively these results are especially interesting since they support the notion that 2A^pro^ activity does not disturb all transport pathways equally, but rather has differential targeting of particular pathways and potentially staggered temporal effects (57). Based on our data, we propose that 2A^pro^ catalytic activity affects Nup98-mediated protein export more significantly than protein import.

**Figure 7:**
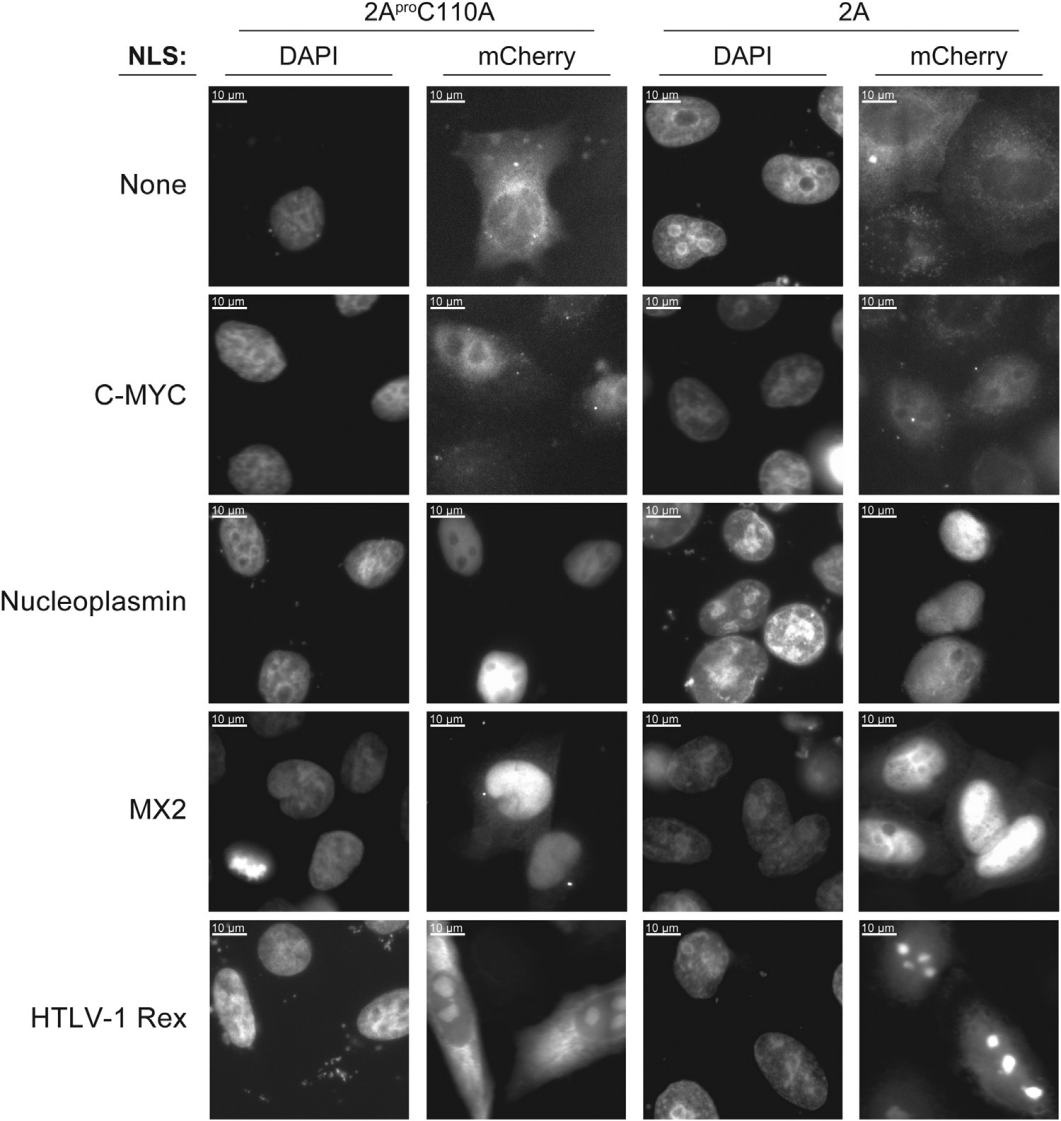
**2A**^**pro**^**-mediated cleavage of Nup98 results in localization changes for transport reporters with various NLS motifs**. Fluorescent reporters consisting of an NLS motif and an mCherry tag, fused to a LacZ peptide. Cellular localization of NLS reporters was assayed with and without 2A^pro^ expression. Nucleoplasmin, MX2, and C-Myc NLS reporters localized to the nucleus, even during 2A^pro^ expression, suggesting those NLS motifs function *via* import pathways are not solely dependent on Nup98. The HTLV-1 Rex NLS motif also has RNA binding activity and shows mislocalization. HTLV-1, human T-cell leukemia virus type 1; NLS, nuclear localization signal; MX2, myxovirus resistance 2 protein.

To further characterize the effects of 2A^pro^ on nucleocytoplasmic transport, we used additional mCherry-tagged NLS reporters incorporated into a LacZ fusion, making the reporter large enough so that it does not suffer from passive diffusion effects (Fig. S12*A*) (60). We assayed c-Myc, nucleoplasmin, myxovirus resistance 2 protein (MX2), and human T-cell leukemia virus type 1 (HTLV-1) Rex NLSs with these reporters. We transiently transfected our HeLa stable cell lines with each NLS reporter as well as a no NLS control and induced protease production to track 2A^pro^-dependent localization changes (Fig. 7). The mCherry control fusion protein lacking an NLS was found in the cytoplasm and was not observed in the nucleoplasm (Fig. 7). This result was expected since the protein lacked a localization signal and was too large to passively diffuse into the nucleus; it also shows that specific 2A^pro^ targeting of Nup98 did not significantly compromise the NPC’s overall permeability barrier properties. Addition of c-Myc NLS, predicted to be imported by importin-α3 (61), localized the reporter to the nucleus, and 2A^pro^ production did not affect its localization. Similarly, the nucleoplasmin NLS localized reporters tightly to the nucleus (60), and 2A^pro^ biosynthesis did not induce relocalization of the reporter, suggesting that nucleoplasmin NLS-dependent import is also not affected by Nup98 degradation. Another NLS was derived from the human MX2. This protein is an interferon-induced inhibitor of HIV-1 infection that localizes to the nuclear envelope and blocks nuclear import of viral cDNAs (60, 62). The N terminus of MX2 contains an NLS-like motif that matches consensus of proline–tyrosine NLS sequences recognized by Kap-β2, adding yet another type of NLS signal to our repertoire of reporters (63). As expected, MX2 NLS-tagged reporters localized to the nucleus, and subsequent 2A^pro^ gene expression failed to significantly affect NLS localization.

The final NLS tested originated from HTLV-1 Rex protein, a viral protein produced during infection. Rex is a regulatory protein that promotes viral replication by exporting viral mRNA from the nucleus to the cytoplasm, where it is translated to produce HTLV-1 proteins (64). The HTLV-1 Rex protein retains an NLS motif that localizes it to the nucleus, but the same NLS motif functions as an RNA binding domain for the Rex-responsive element (RxRE) located at the 3′ end of HTLV-1 mRNAs, allowing it to be re-exported (65). As expected, the Rex NLS-tagged reporter was observed in the cytosol, nucleus, and nucleolus in cells expressing 2A^pro^C110A (60). However, in cells expressing active 2A^pro^, the reporter localizes exclusively to the nucleus and nucleolus. Given the Rex NLS’s dual function, these results can be rationalized by the Rex NLS reporter being normally shuttled between nucleus (and nucleolus) and cytoplasm by virtue of NLS-mediated import and then binding to exporting mRNAs. 2A^pro^ production however diminishes mRNA-mediated export, re-localizing the reporter more predominantly to the nucleus.

In sum, our data indicate that CVB3 2A^pro^ targets and specifically cleaves Nup98 to alter select, predominantly export-related, nucleocytoplasmic transport pathways. Thwarting export of various proteins and mRNAs likely prevents infected cells from mounting a response to picornavirus infection through interferon responses. Our data further underscore how Nup98-mediated export is a convergent target among different viruses, with VSV (66), SARS-CoV-2 (43, 51, 52), and influenza viruses (67) all evolving unique ways to sabotage these transport pathways, likely for similar reasons.

## Discussion

### Protease 2A preferentially binds Nup98 and directly cleaves it

Affinity capture experiments and MS analyses revealed that Nup98 and the eIF3 complex, especially eIF3L, were the predominant interacting partners of 2A^pro^ (Figs. 1*B* and S13). While eIF3L is involved in translation initiation (33, 38), Nup98 is a key player in nucleocytoplasmic trafficking of proteins and mRNA (12, 31, 40, 55, 58, 68). Nup98 is a mobile FG Nup, detected on both cytoplasmic and nuclear sides of the NPC. Nup98 forms tight binding interactions with Rae1 through its GLEBS motifs, facilitating Rae1 localization to the NPC where it serves as an mRNA export factor (31, 32). Our results imply that Rae1 is not itself a binding target for 2A^pro^; instead, they indicate that 2A^pro^ specifically binds to and then cleaves Nup98, resulting in loss of both Nup98 and Rae1 from the NPC.

### Protease 2A likely interacts with eIF3L to facilitate target selection and eIF4G cleavage

Eukaryotic translation initiation depends on initiation factors, such as eIF4G, eIF4E, as well as 40S ribosomal subunit bound to eIF3 (39, 69, 70). These factors form scaffolds that facilitate correct ribosomal attachment to mRNA and loading of tRNAs (71), as well as form focal points for cellular regulation of translation (72, 73). Based on our findings, we hypothesized that eIF4G cleavage was mediated through a different mechanism than Nup98 cleavage—*i.e.*, rather than binding directly to its cleavage target eIF4G, it interacts *via* a second order interaction, with the eIF3 complex forming the first order binding interaction to 2A^pro^. We thus propose a novel cleavage mechanism—*i.e.*, 2A^pro^ binds the eIF3 complex by interacting directly with eIF3L and possibly with other eIF3 components, with these interactions constituting a platform from where 2A^pro^ can cleave eIF4G during translation initiation (Figs. S6*C* and 8).

**Figure 8:**
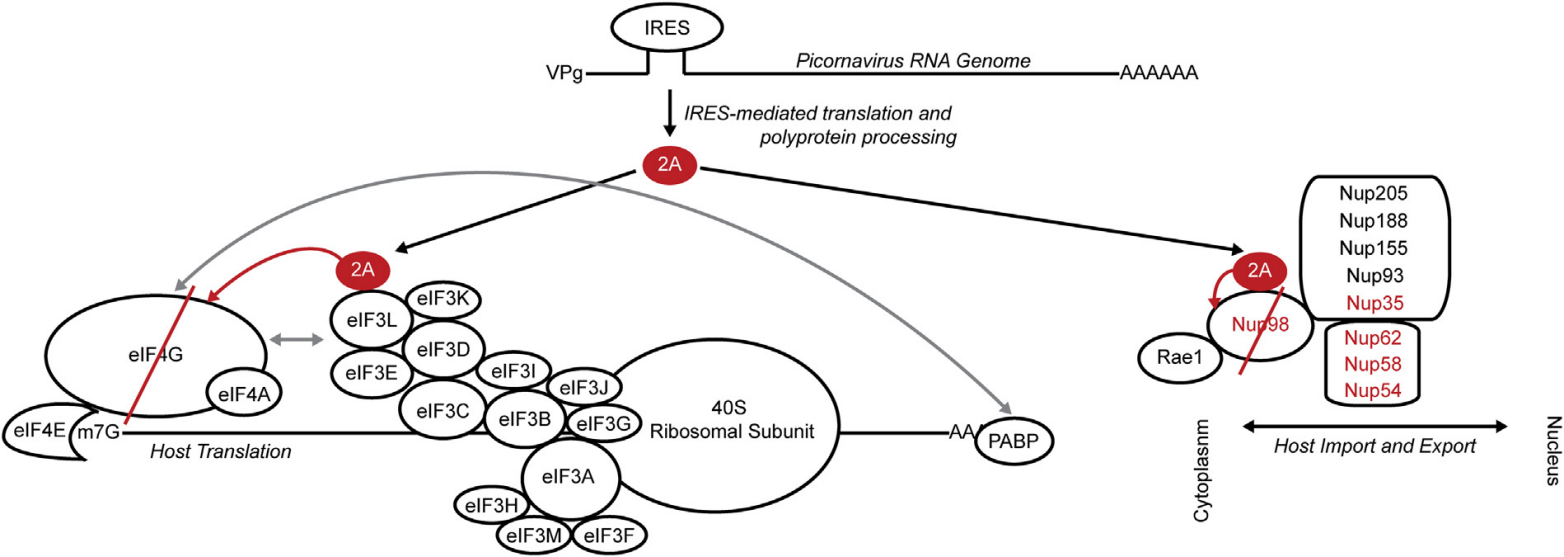
**Unified model of initial stages of picornaviral protease expression and function**. Upon virion intake and genome release, the picornaviral RNA genome is translated as a polyprotein that is proteolytically processed into individual protein subunits by proteases 2A and 3C. The 2A protease proceeds to cleave Nup98 and eIF4G, with the latter cleavage facilitated by 2A^pro^ association with the eIF3 complex. Cleavage of Nup98 dissociates Rae1 from the NPC, and the loss of both proteins from the NPC impacts nuclear transport of proteins and mRNA and relocalizes nuclear factors that promote internal ribosome entry site (IRES)-mediated translation. In parallel, eIF4G cleavage results in a shutdown of the host cell’s translation capability, diminishing the infected cells’ ability to react to the picornaviral infection. As the infection continues and additional viral proteins are translated, 2A^pro^ activity results in dozens of secondary and tertiary cleavage targets, such as Matrin3, MAVS, and PABP, among many others (28). NPC, nuclear pore complex.

### Protease 2A-mediated degradation of Nup98 and eIF4G are among the first host cleavage events that occur during picornaviral infection

Picornaviral proteases, particularly 2A^pro^, from multiple strains such as CVB3 and poliovirus are reported to engage cellular targets early during infection (27, 28). Protease production during picornaviral infection can be measured by cleavage of established targets such as eIF4G. As early as 2 h postinfection, one can detect eIF4G and Nup98 cleavage during RV (27, 74) and CVB3 infections (28). From an evolutionary perspective, shutting down host translation and abrogating steady-state nuclear import and export through Nup cleavage could prevent a mounted innate immune response by decreasing import of immune signaling proteins, such as STAT1/2 (43, 66), and in particular by preventing biosynthesis of new proteins in response to signaling cascades. Despite the swiftness with which 2A^pro^ targets eIF4G and Nup98, the vast majority of other characterized 2A^pro^ cleavage targets are not degraded until much later, when picornavirus protein production and replication within cells is occurring at an exponential pace (28). Such precise targeting of Nup98 and eIF4G has to be facilitated by a general 2A^pro^ “preferred affinity” for those targets. Altogether, we identified a strong affinity of 2A^pro^C110A for Nup98 and eIF3L during affinity captures, which further supports our overarching hypothesis that the 2A^pro^ specifically targets mainly Nup98 and eIF4G during the initial stages of picornavirus infection.

### Protease 2A alters mRNA localization

Nup98 is a critical player in protein transport and mRNA export. Interfering with Nup98-Rae1 function is not a unique viral strategy for controlling host RNA trafficking. VSV M protein and SARS-CoV-2 Orf6 both target Nup98-Rae1 to disrupt nuclear transport in infected cells (43, 51, 66, 75). While M protein and Orf6 disrupt normal Nup98 function by directly binding it and Rae1, picornaviruses evolved to cleave Nup98 through the catalytic activity of the 2A^pro^. Our studies revealed a striking difference in mRNA localization between 2A^pro^ and 2A^pro^C110A expressing cells, as visualized by changes in intensity and signal distribution of mRNA ‘speckles’ in response to 2A^pro^ catalytic activity (Fig. 5). Furthermore, our studies revealed colocalization between 2A^pro^-induced granules and G3BP1 signal (Fig. S10), suggesting mRNA may be localizing within stress granules. In parallel, during our cellular fractionation experiments, we observed that depletion of Nup98 through 2A^pro^-mediated cleavage delocalizes Rae1 from the nucleus into the cytosol (Fig. 4). These observations lead us to hypothesize that picornaviruses affect mRNA localization by inducing cellular stress responses and through Nup98 degradation and delocalization of Rae1 (27, 54, 55, 76, 77). This is consistent with published work with M protein- (66) and Orf6-mediated (43) mRNA export interference, reinforcing the observable convergent evolution of RNA viruses on targeting Nup98-Rae1.

### Protease 2A specifically inhibits Nup98-mediated nuclear protein export

Nup98 mediates a number of protein transport pathway by serving as a docking site for Kaps and other transport factors as well as *via* its associated mRNA export factor, Rae1 (68). We demonstrated that 2A^pro^ catalytic activity and cleavage of Nup98 was primarily responsible for affecting nuclear export of various reporters (Fig. S12*B*). Although nuclear import was also affected, the effects were relatively minor compared to the changes observed with nuclear export. Additional major transport changes during picornaviral infection likely occur due to the expression and production of the 3C protease, which has also been shown to cleave NPC components (7), as well as the exponential production of both proteases during infection. Nevertheless, our data reinforce the idea that a primary function of 2A^pro^ is to disrupt efficient nuclear export of host cell proteins as rapidly as possible after viral infection.

### A unified model of protease 2A–mediated cleavage and downstream effects during early stages of infection

Based on published literature and our studies, we formulated a unified model of the initial stages (first 2–3 h) of picornaviral infection, as pertaining to 2A^pro^ (Fig. 8). The picornaviral RNA genome is expressed as a polyprotein and proteolytically processed by 2A^pro^ and 3C^pro^ into individual components. We conclude that prior to exponential viral protein production, 2A^pro^ specifically targets eIF4G (likely by binding to the adjacent eIF3L) and Nup98, key players in regulating critical functions of host cells, rapidly destroying both proteins. eIF4G cleavage results in an arrest of host translation, preventing accumulation of new host proteins, and creating eIF4G cleavage products, which partake in internal ribosome entry site-mediated translation of the picornaviral genome and promotion of viral translation (78). Nup98 degradation largely abrogates a number of nuclear export pathways, including cellular mRNA export; together with eIF4G cleavage, these changes attenuate the ability of infected cells to mount an innate immune response. Once the picornaviral infection runs for 4 to 6 h, viral protein production increases exponentially to produce orders of magnitude more 2A^pro^ compared to the earliest time points. This higher concentration of 2A^pro^ then drives the cleavage of secondary and tertiary protein targets, such as MAVS and TRIF, among many others (9, 11, 79). Altogether, these studies advance our understanding of 2A^pro^ functions and provide new insights into the kinetics and consequences of eIF4G and Nup98 cleavage during picornaviral infection.

## Experimental procedures

### Molecular cloning

epB-Puro-TT-CVB3 2A (WT) and 2A^pro^C110A (catalytically inactive mutant) plasmids for PiggyBac stable cell transfection were generously donated from the Rice laboratory. Gibson Assembly (New England Biolabs) was utilized to add an N-terminal GFP tag to the proteases for downstream affinity capture experiments, as per manufacturer specifications. Newly prepared vectors were transformed and amplified in SURE2 Ultracompetent cells, specifically designed for cloning of genes involved in rearrangement and deletion of DNA, improving cloning efficiency for PiggyBac vectors that contain sites for DNA recombination.

The pHR39-CMV-GFP-LacZ plasmid backbone with a diverse array of NLS sequences was graciously shared with us by Melissa Kane (University of Pittsburgh). These vectors were used to prepare new plasmids with the EGFP sequence replaced by an mCherry sequence (from the pRS426-GPD-mCherry-4xMS2 plasmid) for downstream fluorescent imaging during GFP-2A^pro^ gene expression. These mCherry-NLS reporters were also prepared through Gibson Cloning procedure, as per manufacturer specifications.

### Tissue culture

Tissue culture cell lines (HeLa and HEK293T) were maintained and expanded in growth media, Dulbecco's modified Eagle's medium (Invitrogen-11965) with 2 mM L-glutamine (Life Technologies), penicillin–streptomycin (100 U/ml) (Life Technologies), and 10% (v/v) fetal bovine serum (Sigma-Aldrich Cat. # F2442). For transient and stable cell transfections, we followed the JetPrime user protocol (Polyplus). For stable cell transfection with PiggyBac vectors, we also added a transposon expressing vector (System Biosciences), as per manufacturer specifications. The PiggyBac Transposase system randomly integrates a select gene into target cell chromosomal DNA, along with a puromycin resistance gene. Once transfected, stable cells are selectively pressured with puromycin (1 μg/ml) in growth media. Once cells reached an expression time point, cells were collected by trypsin-mediated detachment, washed with Dulbecco's PBS, and prepared for lysis in our affinity capture buffer (300 mM NaCl, 20 mM Hepes, pH. 7.4, 0.5% Triton-X 100.

### Cryogenic lysis

Cryogenic lysis is an effective approach pioneered by the Rout laboratory for large-scale preparation of eukaryotic cells prior to affinity capture or other biochemical assays. Cells are grown in large quantities, collected, and ground to fine powder under −140 °C conditions with a mill and steel balls. This lysis process creates homogeneous cell powder and provides an opportunity to assay a variety of affinity capture conditions. Grams of cryogenically frozen cells are milled in a Teflon jar with steel balls, as per established protocols (80, 81). The resulting powder can be measured out in milligrams for downstream affinity capture experiments.

Affinity capture protocols were established based on existing in-house Rout laboratory protocols. Several different types of buffer conditions (150–500 mM NaCl, 20 mM Hepes, pH 7.4, 0.5% Triton X-100) were assayed before one was chosen for quantitative MS analyses downstream of affinity captures. All lysis were done with protease inhibitor cocktail (Complete-EDTA free) and at +4 °C. Affinity capture was performed with magnetic dynabeads conjugated to GFP-specific nanobodies (developed in-house) and clarified lysates. Captured protein complexes were eluted with lithium dodecyl sulfate or ammonium hydroxide as per previously described protocols (82).

### MS and sample preparation

Affinity-purified reduced and alkylated (iodoacetamide) protein samples were run ∼5 mm into a 10% bis-Tris SDS-polyacrylamide gel (gel plug), and gels were Coomassie blue stained. Stained gel regions were excised, cut into 1 mm cubes, destained, and dehydrated. Ten microliter of 0.1 μg/μl trypsin (Roche) solution was added to the destained gel pieces, which were allowed to rehydrate for 10 min on ice. Forty-five microliter of 25 mM ammonium bicarbonate/10% acetonitrile buffer was then added, and gel pieces were digested overnight at 37 °C. An equal volume of 2.5 mg/ml POROS R2 20 μm beads (Life Technologies #1112906) in 5% v/v formic acid, 0.2% v/v TFA was added, and the mixture incubated on a shaker at 4 °C for 24 h. Peptides were desalted on C18 ZipTips (MilliporeSigma), eluted, and concentrated by vacuum centrifuge to ∼10 μl. Two microliter were injected per LC-MS/MS analysis.

Samples were analyzed with a nano-LC 1200 (Thermo Fisher) using a EASYspray PepMap RSLC C18 3 micron, 100 Å, 75 μm by 15 cm column. GFP pulldown samples were analyzed using an Orbitrap Q Exactive Plus (Thermo Fisher) and separated using a 44 min gradient. The instrument was operated in data-dependent mode, and the 20 most abundant ions were selected in each full scan and sequentially fragmented by high-energy collisional dissociation using a normalized collision energy of 24%; dynamic exclusion was enabled. Target resolution was 70,000 for MS. The quadrupole isolation window was 2.0 m/z, and the MS/MS used a maximum injection time of 100 ms with one microscan and a minimum intensity threshold of 1E4.

Nup214 pulldown samples were analyzed using a Orbitrap Lumos instrument (Thermo Fisher) and separated using a 120 min gradient. The instrument was operated in data-dependent mode, and the most abundant ions were selected in each full scan for the maximum parallelizable time and sequentially fragmented by high-energy collisional dissociation using a normalized collision energy of 28%; dynamic exclusion was enabled. Target resolution was 120,000 for MS. The quadrupole isolation window was 1.4 m/z, and the MS/MS used a maximum injection time of 250 ms with one microscan and a minimum intensity threshold of 5E3.

### Proteomic data analysis

MS data files were converted to mgf format and searched against a human proteome database and an in-house generated protein database including the bait proteins (GFP, GFP_2A^pro^, or GFP_2A_C110A^pro^) using a standard Sequest HT-Percolator workflow in Proteome Discoverer 2.1 (Thermo Fisher). Searches and label-free quantitation were conducted using parameters specified (Table S7).

Data output was generated in CSV format and converted to an Excel sheet for curation and analysis. Proteins with fewer than three unique peptides were removed. The datasets were curated by a list of nonspecific GFP interactors identified in the CRAPome database (Table S6). The CRAPome contaminants list was developed from multiple submitted GFP affinity capture experiments. The remaining proteins are enriched during affinity capture and represent potential interacting partners of the handle. Final data analysis included averaging proteins identified in three datasets and normalizing by protein abundance (Table S1). The Nup214 MS analysis pipeline was based on the 2A^pro^ protocol above. Nup214 pulldown datasets were averaged and interrogated against the CRAPome contaminants list. Datasets were normalized by protein abundance, before plotting the fold difference of 2A^pro^C110A:2A^pro^ (transformed by Log_2_) over 2A^pro^C110A protein abundance (transformed by Log_10_) (Table S2).

Three technical replicates were conducted of each sample in each pulldown, and variation between technical replicate measurements is indicated by error bars in the respective figures. Variation in abundance of the bait protein (GFP, GFP_2A^pro^, or GFP_2A_C110A^pro^, or Nup214) was ∼25% between replicates, in line with comparable studies using label free quantitation (83, 84).

### Immunofluorescence and immunostaining of mammalian cell lines

We assayed stable HeLa cell lines constitutively expressing GFP_2_-NLS/NES fusion proteins to validate published literature describing 2A^pro^-mediated relocalization of such reporters with transient transfection of GFP-2A^pro^ and GFP-2A^pro^C110A. Note that while both sets of signals are “GFP” and emit light on the same wavelength, the GFP_2_ reporters are enhanced GFP (eGFP), while the proteases’ tagged GFP is the original fluorescent protein. The eGFP signal output is over 50-fold greater than regular GFP (85). In addition, GFP_2_ reporters have twice as much signal (two copies of eGFP), with tagged proteases retaining a single copy. Side-by-side comparisons demonstrated GFP production did not interfere with the eGFP reporter protein signals (data not shown), thus allowing us to use these validated reporters.

Cells are washed with PBS, fixed in 4% paraformaldehyde (PFA) at room temp for 15 min, washed with PBS to remove excess PFA, permeabilized using 0.2% Triton-X 100 in PBS for 10 min, followed by one more set of washes. Fixed and permeabilized cells are blocked with 5% goat serum, 1% BSA, in PBS at room temperature for 1 h. Primary antibodies are incubated with blocking solution at +4 °C overnight. Cells are incubated with secondary antibody in PBS at room temperature for 2 h. Nuclei are stained with DAPI in PBS at room temperature for 10 min. Coverslips are mounted with ProLong Gold Antifade. Fluorescent images were obtained with the Discover Echo Revolve Fluorescence Microscope with the 60x objective.

### Fluorescent reporters to assay 2A^pro^ effects on nuclear transport

Fluorescently tagged (GFP or mCherry tags) fusion proteins with localization tags were developed by the Rout laboratory as reporters to study nuclear-cytoplasmic transport. These reporters are useful for a number of biological contexts: for instance, at over- or under-production of various Nups implicated in transport, during addition of chemical blockers of transport or during expression of viral protein genes implicated in affecting transport. In our case, we were keen on exploring the effects of the 2A^pro^ on nuclear-cytoplasmic trafficking.

### Poly-T FISH staining for assaying protease-mediated effects on mRNA localization

Buffers: 20x SSC buffer—3M NaCl, 400 mM Na-citrate. Hybridization buffer—1 mg/ml yeast tRNA, 0.005% goat serum, 10% dextran sulfate, 25% formamide, 2x SSC.

Oligo-dT(30) conjugated with Tye563 were purchased from IDT and used to bind mRNA. Cells were washed, fixed with 4% PFA, permeabilized with 0.2% Triton X-100 in PBS, and washed again prior to FISH. Cells were stained with 100 nM Oligo-dT in hybridization buffer at +37 °C in a humidity chamber overnight. Cells were washed with 4x SSC prewarmed to +37 °C, then with 2x SSC prewarmed to +37 °C, and finally twice with 2x SSC at room temperature. Cells were stained with DAPI and mounted as previously described.

### Poly-T FISH fluorescence quantitation

Fluorescence images and data were quantitated with ImageJ (Fiji 1.51f). Signal levels were captured using the rectangular window tool, with small squares measuring mean density in nuclear and cytoplasmic portions of cells. Signal means were normalized by background, before calculation of ratios of nuclear to cytoplasmic signal. The ratio of nuclear:cytoplasmic signal in GFP expressing cells was set as the control, with respective ratios during 2A^pro^ and 2A^pro^C110A expression evaluated for increase or decrease of nuclear signal. Standard deviations were calculated based on averages of nuclear:cytoplasmic ratios.

### Stable cell lines constitutively expressing NLS- or NES-tagged fluorescent reporters

The Rout laboratory developed stable cell lines constitutively expressing GFP-GFP–NLS/NES fusion proteins. Stable lines with HeLa cells expressing GFP_2_-NLS/NES/NLS–NES protein fusions were prepared. The NLS sequence was cloned from SV40 Large T-antigen, the NES peptide sequence was from PKI, and the NLS–NES was the combination of both. Reporters would localize to their respective compartments, with the NLS–NES fusion mostly localizing to the cytoplasm. Any external stress, such as 2A^pro^ expression, would relocalize reporters to a new compartment accordingly. Stable cell lines constitutively expressing fluorescent reporters were transiently transfected with 2A^pro^ or 2A^pro^C110A, as per established protocol. Cells were subsequently fixed with formaldehyde and treated as per immunostaining protocol.

### Fluorescent transport reporter quantitation

Fluorescence images of nuclear/cytoplasmic reporters were quantitated as previously described for Poly-T FISH. Mean density was measured in nuclear and cytoplasmic fractions, normalized by background, and sample nuclear:cytoplasmic ratios were evaluated for differences during 2A^pro^ and 2A^pro^C110A expression. Standard deviations were calculated as previously described.

### HeLa cells expressing NLS-tagged fluorescent-LacZ fusion reporters

NLS/NES-mCherry-LacZ fusion reporters were also developed to study nuclear-cytoplasmic trafficking. These fusion proteins are considerably larger than most assayed NLS/NES reporters and as such, should not be affected by passive transport effects. We assayed these reporters by transiently transfecting them into HeLa stable cell lines with integrated GFP/GFP-2A^pro^/GFP-2A^pro^C110A, 12 to 16 h before induction of protease expression. Cells produced protease for 12 to 16 h before being fixed with formaldehyde and treated as per immunostaining protocol.

### Biochemical fractionation of mammalian cell lines into nuclear and cytoplasmic fractions

Cell fractionation was performed by using modifications (Udi *et al*. manuscript in preparation) of methods in the published literature (50).

### XTT assay for cell viability and proliferation

The cell proliferation assay was preformed using the Invitrogen XTT (2,3-Bis-(2-Methoxy-4-Nitro-5-Sulfophenyl)-2H-Tetrazolium-5-Carboxanilide) (Product No. X6493) and PMS (Phenazine methosulfate, 98%) (Product No. AC130160010). XTT is a colorimetric assay for cell viability and cytotoxicity. We followed the provided user guide for execution of this experiment.

## Data availability

Raw data files and sequence databases used are available on the Zenodo repository as “LC-MS raw data for proteomic elucidation of the targets and primary functions of picornavirus 2A protease,” https://doi.org/10.5281/zenodo.6109742.

## Supporting information

This article contains supporting information.

## Conflict of interest

The authors declare that they have no conflicts of interest with the contents of this article.

## Supporting information

Supplemental Table S7

Supplemental Figures S1–S13 **Figure S1: Western Blot analysis of lysates from HeLa stable cell lines expressing 2A and 2AC110A**. Stable HeLa cell lines were induced to express GFP-, -2A^pro^ and -2A^pro^C110A, with cells collected 16 hours post induction. Cells were lysed in the same buffer conditions as our immunoprecipitation experiments, and the clarified lysates were subsequently analyzed by immunoblot. We assayed a number of proteins of interest, with a focus on whether protein levels were depleted during 2A^pro^ expression. Red arrows indicate degradation of proteins. **Figure S2. Immunoblot analysis of cryomilled powder**. Cell powder prepared for our HeLa stable cell lines was lysed, clarified, and the supernatant analyzed by immunoblot for protein expression. We assayed for the presence of GFP, as well as cleavage of Nup98 and eIF4G, as indicated by *red arrows*. eIF4G is nearly entirely cleaved, and the cleavage product can be detected *via* immunoblot. Nup98 is completely cleaved in 2A^pro^-expressing cells. **Figure S3. Biochemical isolation of 2A^pro^ interactome and binding specificity to Nup98**. GFP-2A^pro^C110A and GFP-2A^pro^ pulldowns reveal interactions with Nup98 and eIF3 proteins. IP elutions were clarified by SDS-PAGE and analyzed by silver stain (top panels) and immunoblotting (bottom panels). Nup214 and FG Nup IPs suggest 2A^pro^ specifically cleaves Nup98. Immunoblot analysis suggest that immunopurifying GFP-2A^pro^C110A, also correlates to an enrichment of eIF3L in eluates. **Figure S4. Raw MS data were analyzed with Proteome Discoverer software, which output a protein list of putative interacting partners**. The data were averaged within datasets, ranked by unique peptides, and proteins with fewer than three unique peptides were removed. The remaining data were normalized by the GFP dataset (raw peptide quantities were subtracted) and curated with the CRAPome contaminants list29. The CRAPome database enables contaminant searches by affinity capture tag and experiments, enabling a nonbiased and automated way to filter immunoprecipitation data. The final data were ready for analysis such as rankings by peptide quantity or fold-enrichments. **Figure S5. Fold enrichment of 2A^pro^C110A over 2A^pro^ proteins**. We plotted proteins by fold enrichment (label free MS peptide quantity of 2A^pro^C110A/2A^pro^ datasets) and transformed the final enrichment by Log_2_. The top 20 most enriched proteins in the 2A^pro^C110A dataset are almost entirely NPC and eIF3 components. **Figure S6. Types of 2A^pro^C110A interacting partners**. Based on proteomic data analysis, we started formulating soft definitions for the various types of interacting partners identified in our pulldowns. *A*, Nup98 is a known cleavage target and is predicted to be a direct interacting partner of 2A^pro^C110A. *B*, Rae1 is a strong binding partner of Nup98 and is likely an indirect interactor of 2A^pro^C110A during immunoprecipitation experiments. *C*, the eIF3 complex is predicted to be a targeting platform for 2A^pro^-mediated cleavage of eIF4G. While eIF3L is a likely binder of 2A^pro^ in this scenario, it is also plausible other eIF3 complex proteins play a role in this potentially indirect interaction. **Figure S7. Localization of GFP, GFP-2A^pro^, and GFP-2A^pro^C110A**. Proteins of interest are inducibly expressed in HeLa stable cell lines, and cells are imaged under GFP fluorescence (as live cells). GFP signal distributes evenly across a cell. GFP-2A^pro^ forms small speckles that may be P-bodies or stress granules. GFP-2A^pro^C110A localizes to the cytoplasm and enriches on the rim of the nucleus, presumably *via* Nup98-2A^pro^C110A interactions. **Figure S8. 2A^pro^-induced apoptosis in HeLa cells**. *A*, GFP, GFP-2A^pro^, and GFP-2A^pro^C110A are inducibly expressed in HeLa stable cell lines; whole cells are collected, lysed with RIPA buffer, and analyzed by Western Blotting. Lysates were probed for presence of apoptosis markers, such as caspase-3 and PARP. The presence of 2A^pro^ and 2A^pro^C110A strongly induced a caspase response, as both proteins are known to trigger cytotoxicity. Due to 2A^pro^’s lower levels of production, the observed cytotoxicity as gauged per apoptotic markers may seem lower, so we conducted a cell viability assay. *B*, HeLa stable cell lines were assayed for cell viability and survival with an XTT assay, where cell survival is measured through absorbance. Cells were induced to express proteins of interest or exposed to paclitaxel, a drug that induces cell death *via* apoptosis and assayed at 16 and 40 hr timepoints. At 16 h, cell death can be observed at similar levels between 2A^pro^-expressing and paclitaxel-treated cells. By 40 h, cells expressing 2A^pro^ are the most depleted (p<0.0001) on par with cells treated with paclitaxel. These data suggest 2A^pro^ and 2A^pro^C110A can induce a caspase response, but only 2A^pro^ production results in major cell death. **Figure S9. Analysis of STAT1+2 localization in response to protease activity**. GFP, GFP-2A^pro^ and GFP-2A^pro^C110A are inducibly expressed in HeLa stable cell lines and cells are fractionated into cytoplasmic and nuclear fractions. The resulting lysates were analyzed by Western Blotting and probed for STAT1, STAT2, and fraction markers GAPDH and H3. GAPDH is a cytosolic marker while H3 is a nuclear marker, which begins to mislocalize to the cytoplasmic fraction during 2A^pro^ expression. STAT1 and STAT2 protein levels were quantitated and compared within respective fractions. While production of either catalytically active and inactive 2A protease resulted in a decrease in both STAT proteins, active 2A^pro^ depleted STAT1 and 2 levels twice as much as 2A^pro^C110A. 2A^pro^ nearly depleted STAT2 in both cell fractions, but only nuclear STAT1. **Figure S10. Stress granule formation and mRNA co-localization during 2A^pro^ expression.** Proteins of interest are inducibly expressed in HeLa stable cell lines. Cells undergo oligo-dT FISH and are probed with G3BP1, a known stress granule marker. In response to 2A^pro^ production, cells form mRNA speckles/granules/P-bodies (Fig. 5). Staining with G3BP1 reveals this stress granule marker co-localizing in these “granules of mRNA”, suggesting they may be bona fide stress granules. **Figure S11. GFP signal compared to 2xEGFP signal**. Stable cells lines inducibly express GFP-tagged proteins, which could potentially be problematic during other experiments where NLS reporters are also tagged with GFP (Fig. 6). However, NLS/NES reporters are tagged with 2x EGFPs, which are significantly brighter than WT GFP and expressed at higher levels. Control cells producing GFP were analyzed side by side with cells producing 2xEGFP-NLSNES, collected at identical exposure settings and with brightness levels displayed at two different levels. At 0.1x brightness, GFP signal is virtually undetectable, while EGFP is easily detectable. At 1x brightness, the GFP signal is still barely detectable, while the EGFP signal is extremely strong. NES, nuclear export signal; NLS, nuclear localization signal. **Figure S12. Fluorescent reporters for assaying nuclear import and export**. *A*, we developed fluorescently tagged reporters to assay nuclear import and export in mammalian cell lines. Reporters consist of an NLS motif, an mCherry tag, fused to a LacZ peptide to add mass to the final protein to prevent passive diffusion effects. Stable cell lines were also generated with simpler reporters, featuring a double eGFP with a C-terminal NLS or NES tag. *B*, we assayed six NLS motifs, each had nuclear localization signals as observed in literature, while some had other properties that were explored in our assay. Cellular localization of NLS reporters was schematically represented by cells with and without 2A^pro^ expression. *Red arrows* signify less localization in aforementioned conditions, as mediated by 2A^pro^ activity. Nucleoplasmin, MX2, and C-Myc NLS motifs localized the reporters to the nucleus, even during 2A^pro^ expression, suggesting those NLS motifs function *via* import pathways not solely dependent on Nup98. Other NLS motifs, such as KNS or HTLV-1 Rex, had entirely different functions, such as export (KNS) or RNA binding (HTLV-1 Rex). NES, nuclear export signal; NLS, nuclear localization signal; HTLV-1, human T-cell leukemia virus type 1; MX2, myxovirus resistance 2 protein. **Figure S13. Data compliance and scatter plots of Fig 1B, 2C, and 1C.** Existing data were transformed into scatterplots to display individual entries, means, and standard deviations.
