# Supplementary material for: Proteomic elucidation of the targets and primary functions of the picornavirus 2A protease
Source: J Biol Chem. 2022 Mar 31;298(6):101882. doi: 10.1016/j.jbc.2022.101882 (PMC9168619; doi:10.1016/j.jbc.2022.101882)
Supplement: Supplemental Table S7 [file mmc7.pdf]

Mass spectrometry search parameters:

Name of peaklist-generating software and release version	Sequest HT (2013)
Name of the search engine and release version	Proteome Discoverer 2.1, Sequest HT (2013)
Names of sequence databases searched	Human_Uniprot 042018.fasta, contaminants_database.fasta
Release version/date of sequence database searched	4/20/18
Number of entries in each database actually searched	71349, 252
Specificity of all proteases used to generate peptides	Trypsin (Full): P1: K or R, P1' not P
Number of missed and/or non-specific cleavages permitted	2
List of all fixed modifications (including residue specificity) considered	Carbamidomethyl@C
List of all variable modifications (including residue specificity) considered	Oxidation@M, Acetylation@N-terminus
Mass tolerance for precursor ions	10 ppm
Mass tolerance for fragment ions	0.2 Da, 0.6 Da
Threshold score/Expectation value for accepting individual spectra	0.01
Estimation of false discovery rate (FDR) and how calculated	0.01, target/decoy (Percolator)
Quantification	Proteome Discoverer LFQ, Topn=3, max fold change 100, normalize to total peptide amount
