# Supplementary material for: Proteomic elucidation of the targets and primary functions of the picornavirus 2A protease
Source: J Biol Chem. 2022 Mar 31;298(6):101882. doi: 10.1016/j.jbc.2022.101882 (PMC9168619; doi:10.1016/j.jbc.2022.101882)
Supplement: Supplemental Figures S1–S13 — Figure S1: Western Blot analysis of lysates from HeLa stable cell lines expressing 2A and 2AC110A. Stable HeLa cell lines were induced to express GFP-, -2Apro and -2AproC110A, with cells collected 16 hours post induction. Cells were lysed in the same buffer conditions as our immunoprecipitation experiments, and the clarified lysates were subsequently analyzed by immunoblot. We assayed a number of proteins of interest, with a focus on whether protein levels were depleted during 2Apro expression. Red arrows indicate degradation of proteins. Figure S2. Immunoblot analysis of cryomilled powder. Cell powder prepared for our HeLa stable cell lines was lysed, clarified, and the supernatant analyzed by immunoblot for protein expression. We assayed for the presence of GFP, as well as cleavage of Nup98 and eIF4G, as indicated by red arrows. eIF4G is nearly entirely cleaved, and the cleavage product can be detected via immunoblot. Nup98 is completely cleaved in 2Apro-expressing cells. Figure S3. Biochemical isolation of 2Apro interactome and binding specificity to Nup98. GFP-2AproC110A and GFP-2Apro pulldowns reveal interactions with Nup98 and eIF3 proteins. IP elutions were clarified by SDS-PAGE and analyzed by silver stain (top panels) and immunoblotting (bottom panels). Nup214 and FG Nup IPs suggest 2Apro specifically cleaves Nup98. Immunoblot analysis suggest that immunopurifying GFP-2AproC110A, also correlates to an enrichment of eIF3L in eluates. Figure S4. Raw MS data were analyzed with Proteome Discoverer software, which output a protein list of putative interacting partners. The data were averaged within datasets, ranked by unique peptides, and proteins with fewer than three unique peptides were removed. The remaining data were normalized by the GFP dataset (raw peptide quantities were subtracted) and curated with the CRAPome contaminants list29. The CRAPome database enables contaminant searches by affinity capture tag and experiments, enabling a nonbiased and automat [file mmc8.pdf]

Proteomic elucidation of the targets and primary functions of picornavirus 2A protease

Artem A. Serganov, Yael Udi, Milana E. Stein, Valay Patel, Peter C. Fridy, Charles M. Rice, Mohsan Saeed, Erica Y. Jacobs, Brian T. Chait, Michael P. Rout

Contents:
Supporting Figures 1-13

Figure S1

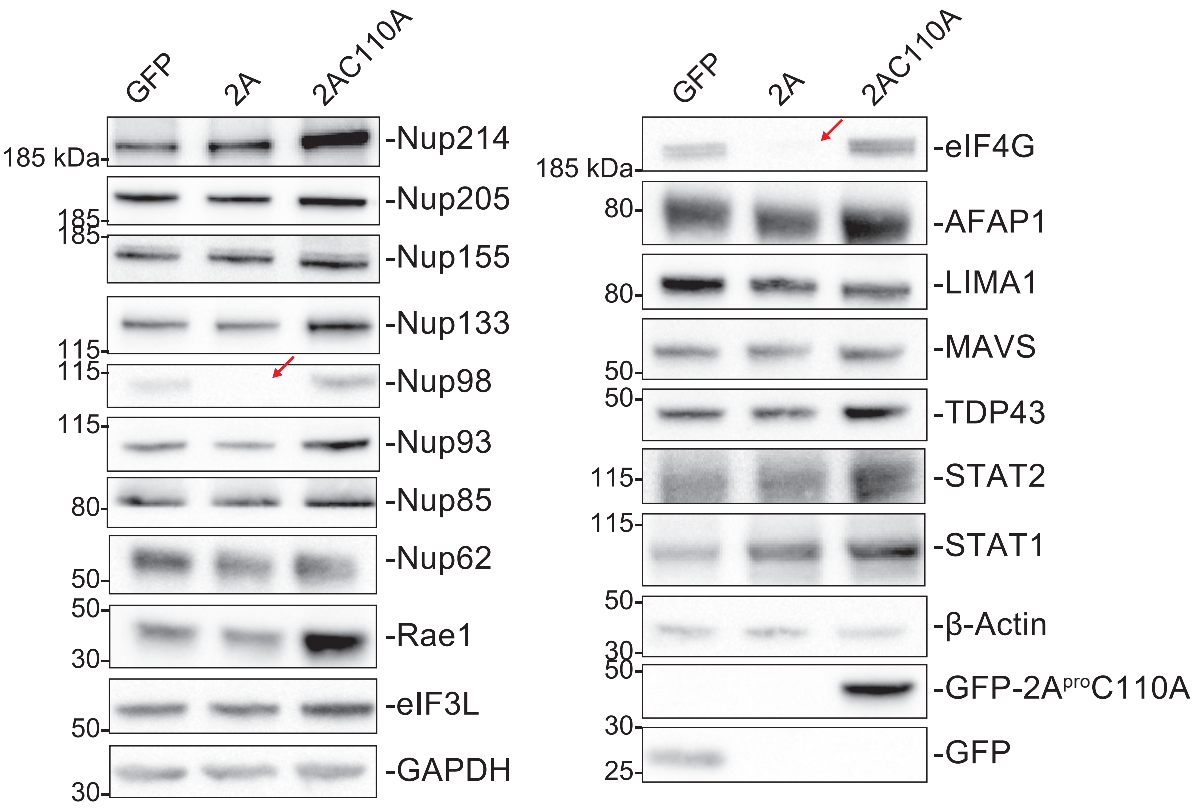
GFP
2A
2AC110A
185 kDa
-Nup214
185
-Nup205
185
-Nup155
115
-Nup133
115
-Nup98
115
-Nup93
80
-Nup85
50
-Nup62
50
30
-Rae1
50
-eIF3L
30
-GAPDH
GFP
2A
2AC110A
185 kDa
-eIF4G
80
-AFAP1
80
-LIMA1
50
-MAVS
50
-TDP43
115
-STAT2
115
-STAT1
50
30
-β-Actin
50
30
-GFP-2A^pro C110A
25
-GFP

Figure S2

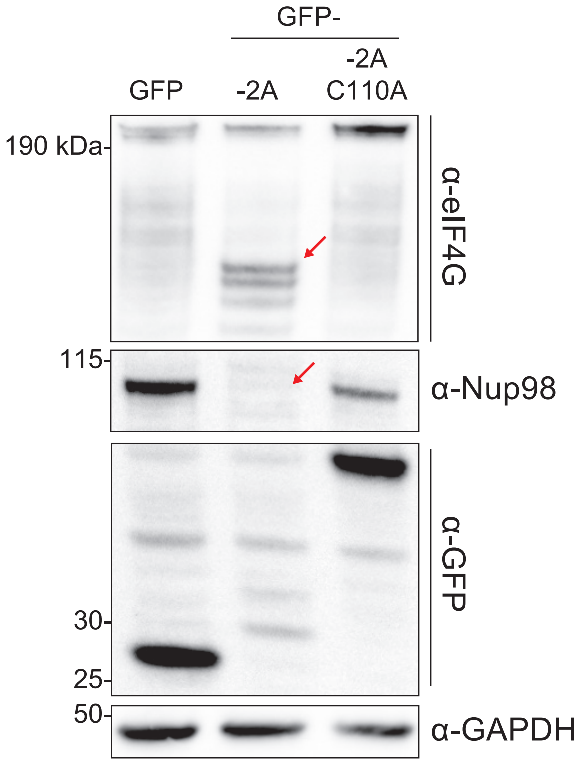
GFP-
GFP
-2A
-2A C110A
190 kDa
115
30
25
50
α-eIF4G
α-Nup98
α-GFP
α-GAPDH

Figure S3

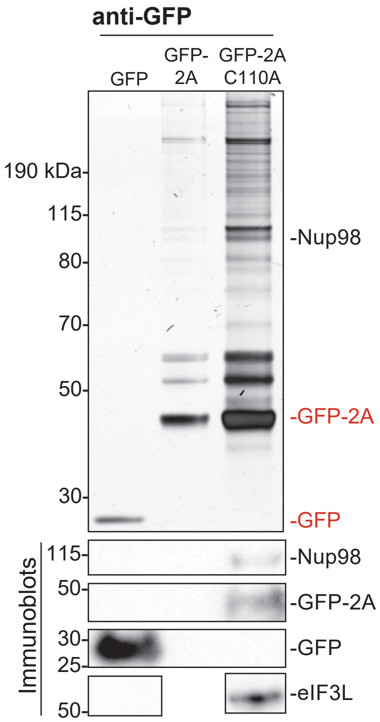

anti-GFP
GFP
GFP-2A
GFP-2A C110A
190 kDa
115
80
70
50
30
-Nup98
-GFP-2A
-GFP
Immunoblots
115
50
30
25
50
-Nup98
-GFP-2A
-GFP
-eIF3L

Figure S4

Raw MS Data (GFP/GFP-2A^{pro}/GFP-2AC110A), n=3

↓

Dataset averaging

↓

Removal of proteins with ≤3 unique peptides

↓

Data normalization to GFP control
GFP-2A^{pro} - GFP
GFP-2AC110A - GFP

↓

CRAPome contaminants curation (54% of list removed)

↓

Data analysis
(1) Rank by peptide quantity
(2) Rank by fold enrichment

Figure S5

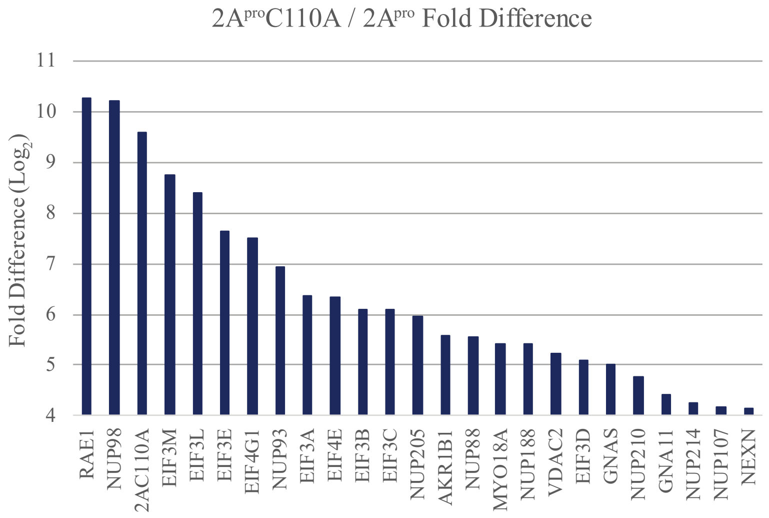
2A^pro C110A / 2A^pro Fold Difference
Fold Difference (Log2)
11
10
9
8
7
6
5
4
RAE1
NUP98
2AC110A
EIF3M
EIF3L
EIF3E
EIF4G1
NUP93
EIF3A
EIF4E
EIF3B
EIF3C
NUP205
AKR1B1
NUP88
MYO18A
NUP188
VDAC2
EIF3D
GNAS
NUP210
GNA11
NUP214
NUP107
NEXN

Figure S6

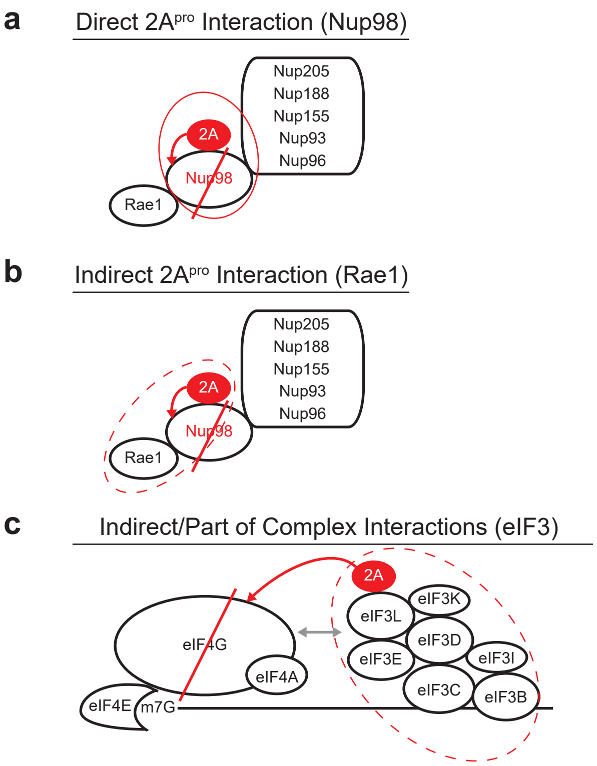
a
Direct 2Apro Interaction (Nup98)
Nup205
Nup188
Nup155
Nup93
Nup96
2A
Nup98
Rae1
b
Indirect 2Apro Interaction (Rae1)
Nup205
Nup188
Nup155
Nup93
Nup96
2A
Nup98
Rae1
c
Indirect/Part of Complex Interactions (eIF3)
2A
eIF3K
eIF3L
eIF3D
eIF3E
eIF3I
eIF3C
eIF3B
eIF4G
eIF4A
eIF4E
m7G

Figure S7

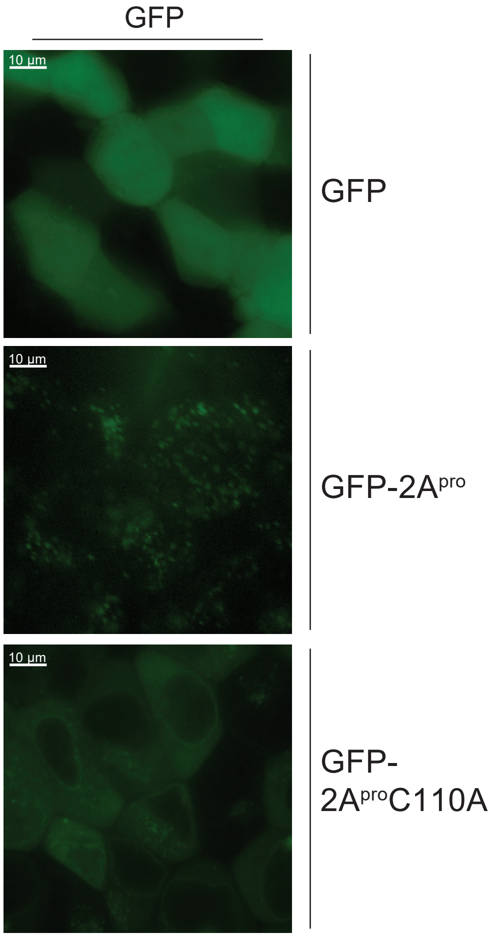

Figure S8

a

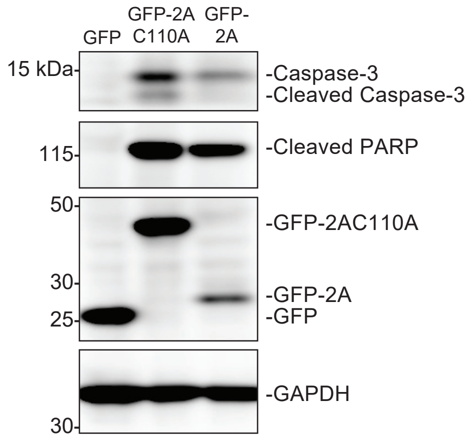
GFP
GFP-2A C110A
GFP-2A
15 kDa
115
50
30
25
30
-Caspase-3
-Cleaved Caspase-3
-Cleaved PARP
-GFP-2AC110A
-GFP-2A
-GFP
-GAPDH

b

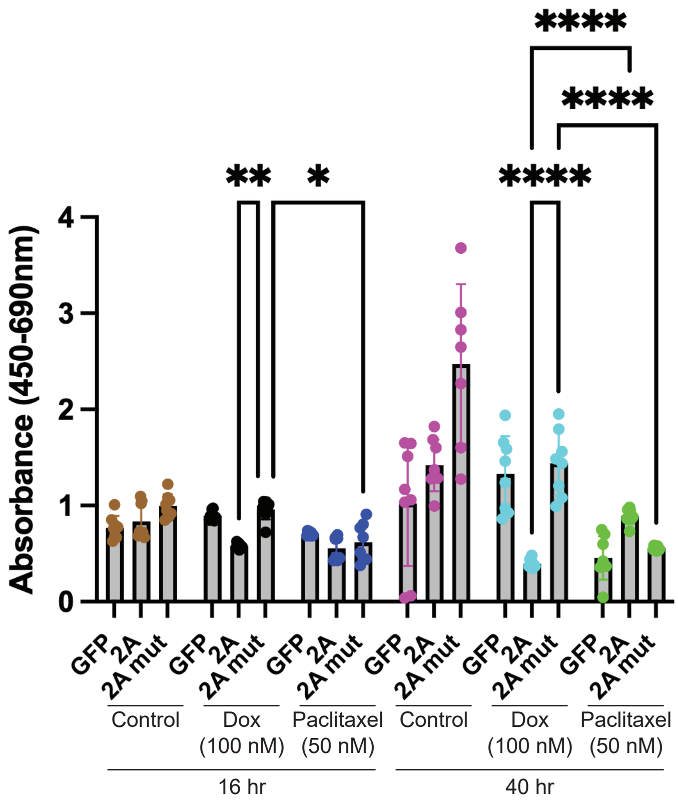
****
****
**
*
****
Absorbance (450-690nm)
0
1
2
3
4
GFP
2A
2A mut
Control
Dox (100 nM)
Paclitaxel (50 nM)
16 hr
40 hr

Figure S9

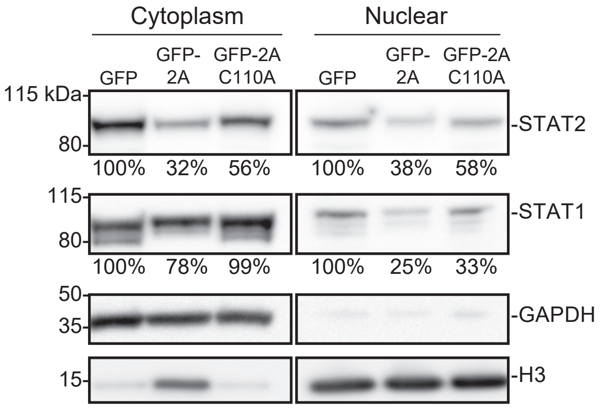

Cytoplasm
Nuclear
GFP
GFP-2A
GFP-2A C110A
GFP
GFP-2A
GFP-2A C110A
115 kDa-
80-
-STAT2
100% 32% 56% 100% 38% 58%
115-
80-
-STAT1
100% 78% 99% 100% 25% 33%
50-
35-
-GAPDH
15-
-H3

Figure S10

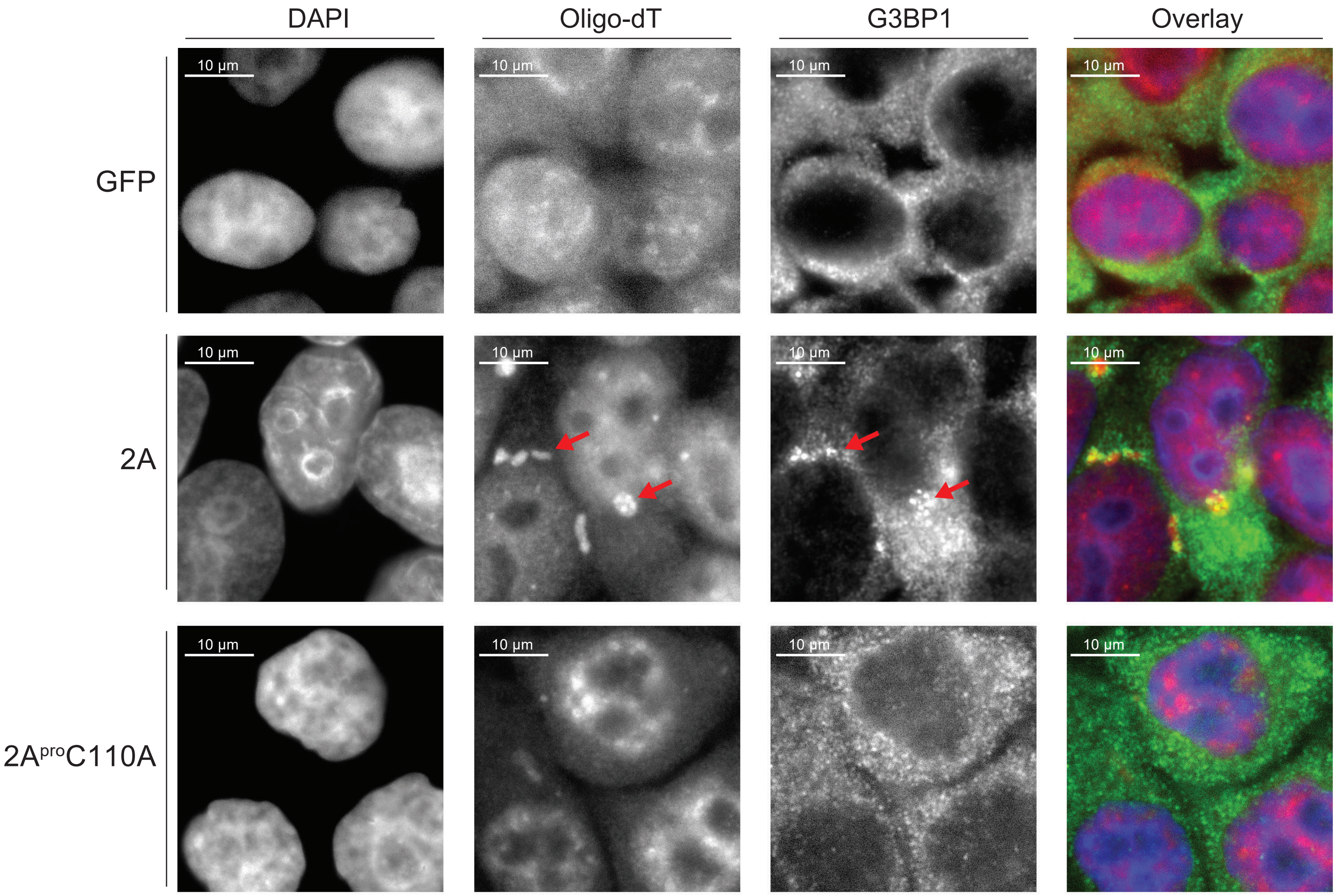

Figure S11

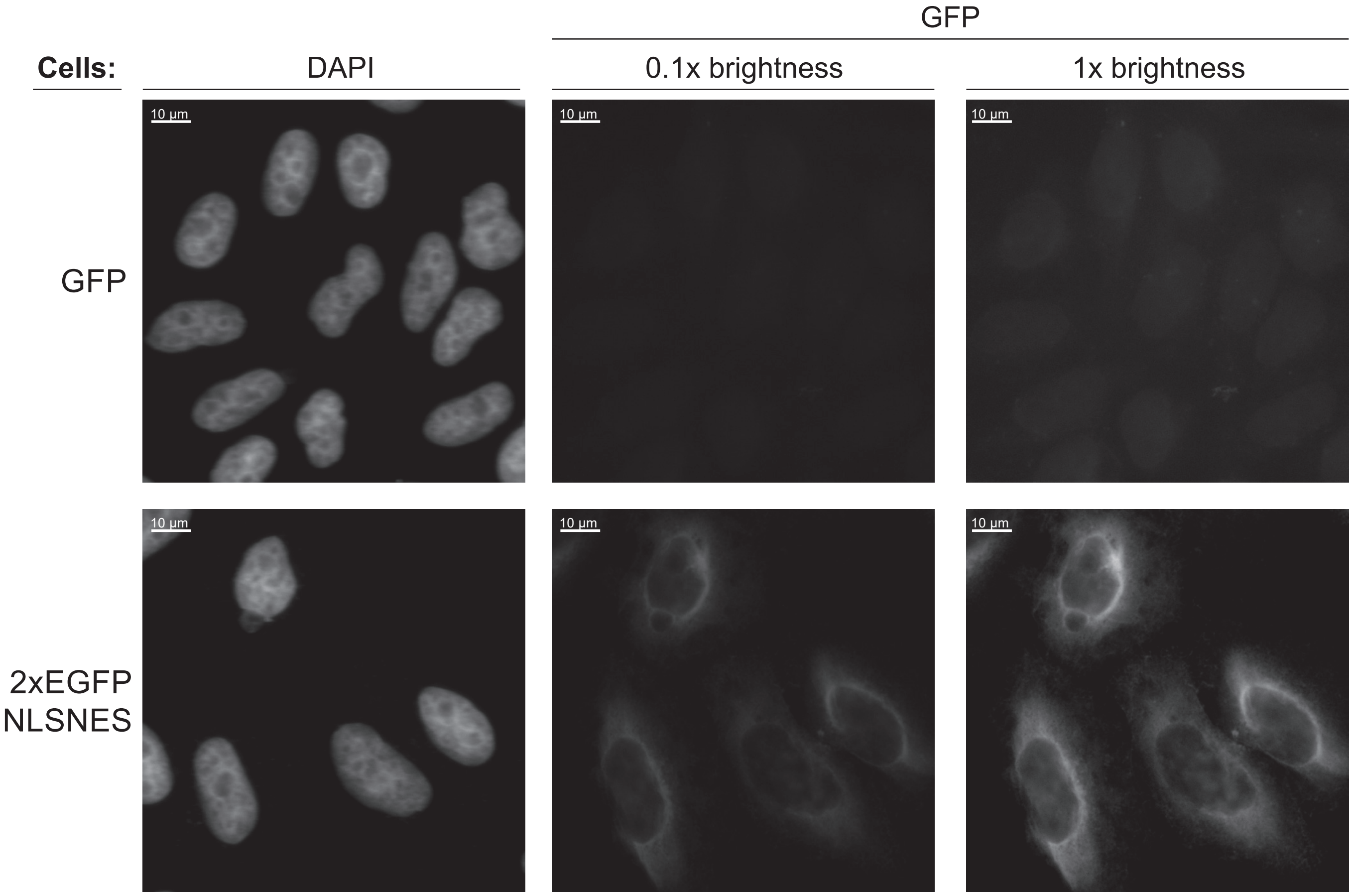
GFP
Cells:
DAPI
0.1x brightness
1x brightness
GFP
2xEGFP NLSNES
10 μm

Figure S12

a

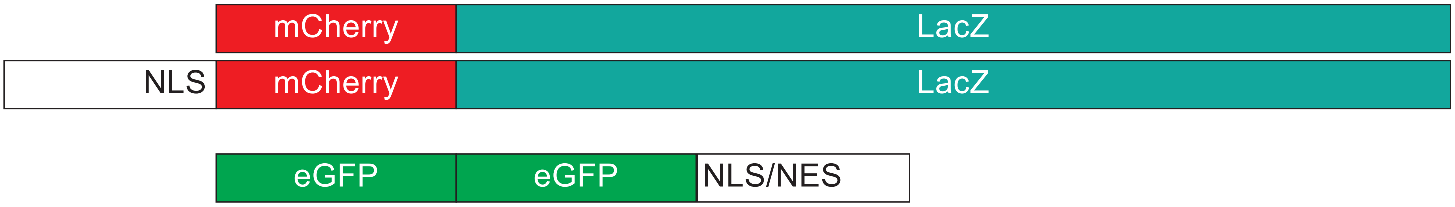

b

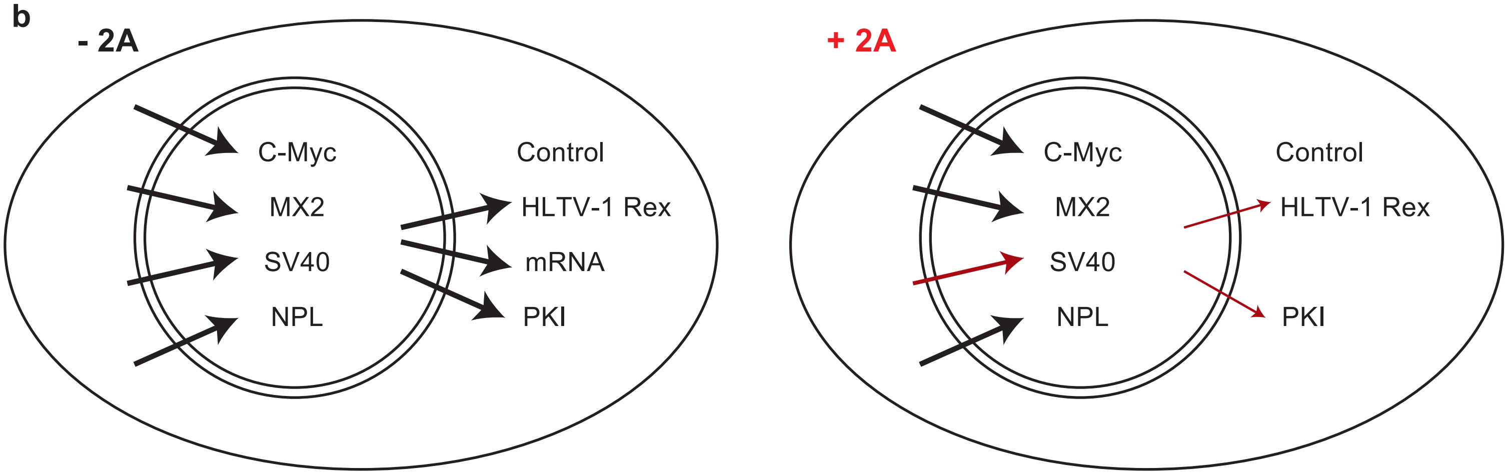

Figure S13

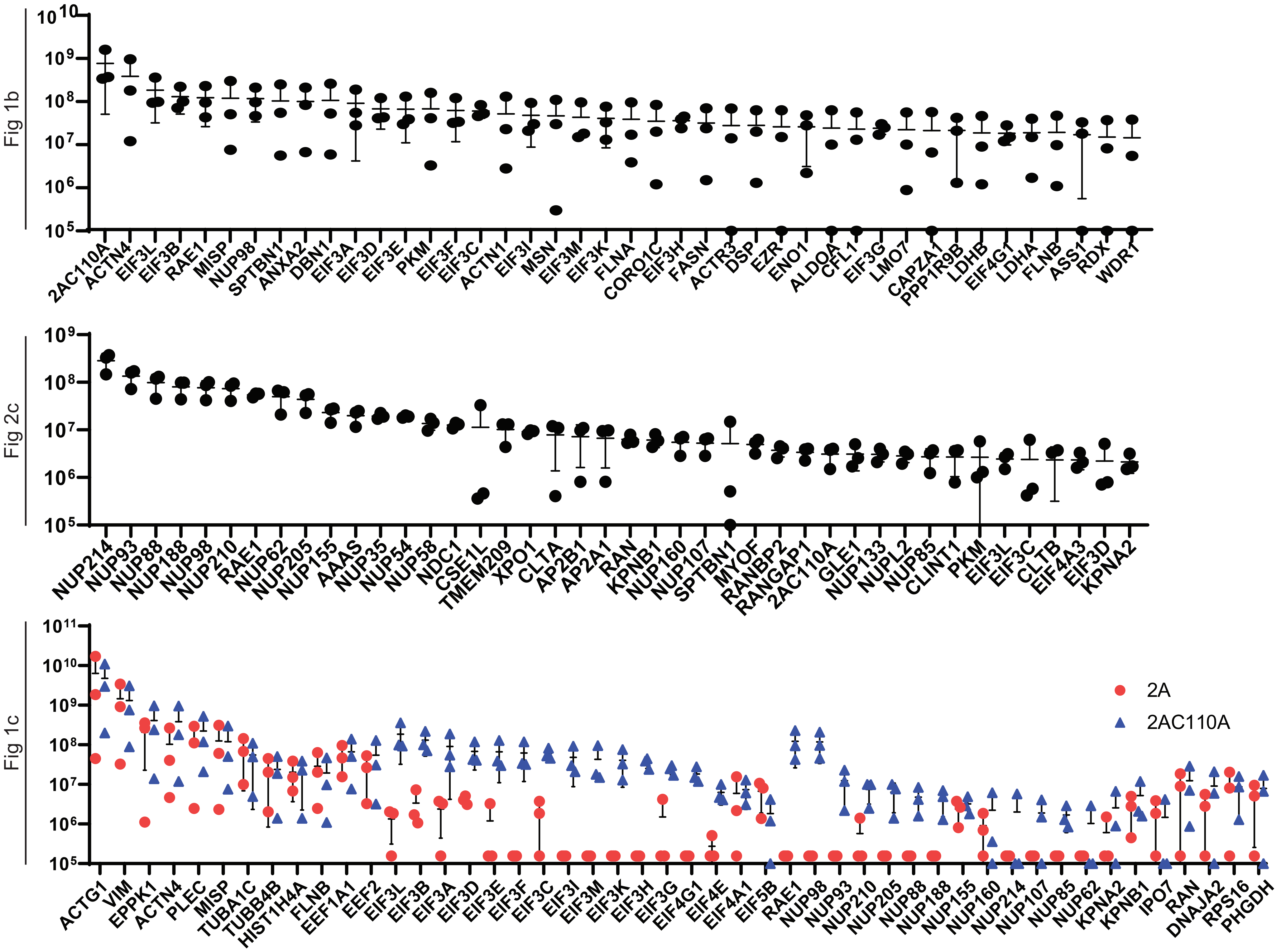
Fig 1b
2AC110A ACTN4 EIF3L EIF3B RAE1 MISP NUP98 SPTBN1 ANXA2 DBN1 EIF3A EIF3D EIF3E PKM EIF3F EIF3C ACTN1 EIF3I MSN EIF3M EIF3K FLNA CORO1C EIF3H FASN ACTR3 DSP EZR ENO1 ALDOA CFL1 EIF3G LMO7 CAPZA1 PPP1R9B LDHB EIF4G1 LDHA FLNB ASS1 RDX WDR1
Fig 2c
NUP214 NUP93 NUP88 NUP188 NUP98 NUP210 RAE1 NUP62 NUP205 NUP155 AAAS NUP35 NUP54 NUP58 NDC1 CSE1L TMEM209 XPO1 CLTA AP2B1 AP2A1 RAN KPNB1 NUP160 NUP107 SPTBN1 MYOF RANBP2 RANGAP1 2AC110A GLE1 NUP133 NUPL2 NUP85 CLINT1 PKM EIF3L EIF3C CLTB EIF4A3 EIF3D KPNA2
Fig 1c
2A
2AC110A
ACTG1 VIM EPPK1 ACTN4 PLEC MISP TUBA1C TUBB4B HIST1H4A FLNB EEF1A1 EEF2 EIF3L EIF3B EIF3A EIF3D EIF3E EIF3F EIF3C EIF3I EIF3M EIF3K EIF3H EIF3G EIF4G1 EIF4E EIF4A1 EIF5B RAE1 NUP98 NUP93 NUP210 NUP205 NUP88 NUP188 NUP155 NUP160 NUP214 NUP107 NUP85 NUP62 KPNA2 KPNB1 IPO7 RAN DNAJA2 RPS16 PHGDH
